# Isolation and synthesis of cryptosanguinolentine (isocryptolepine), a naturally-occurring bioactive indoloquinoline alkaloid

**DOI:** 10.1039/d0ra03096a

**Published:** 2020-05-19

**Authors:** Elida N. Thobokholt, Enrique L. Larghi, Andrea B. J. Bracca, Teodoro S. Kaufman

**Affiliations:** Instituto de Química Rosario (IQUIR, CONICET-UNR), Facultad de Ciencias Bioquímicas y Farmacéuticas, Universidad Nacional de Rosario Suipacha 531 S2002LRK Rosario Argentina kaufman@iquir-conicet.gov.ar bracca@iquir-conicet.gov.ar +54-341-4370477 +54-341-4370477

## Abstract

Cryptosanguinolentine (isocryptolepine) is one of the minor naturally-occurring monomeric indoloquinoline alkaloids, isolated from the West African climbing shrub *Cryptolepis sanguinolenta*. The natural product displays such a simple and unique skeleton, which chemists became interested in well before it was found in Nature. Because of its structure and biological activity, the natural product has been targeted for synthesis on numerous occasions, employing a wide range of different strategies. Hence, discussed here are aspects related to the isolation of isocryptolepine, as well as the various approaches toward its total synthesis.

## Abstract 2

The isolation and properties of the heterocycle are detailed and the diversity of chemical approaches toward the natural product are systematically ordered and reviewed.

## Graphical abstract

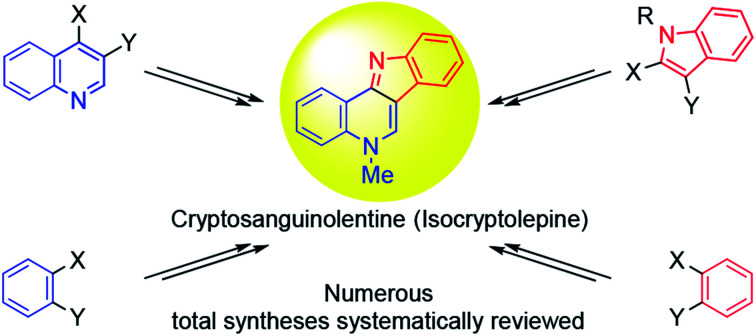

## 1. Introduction

It is now common knowledge that after millions of years of co-existing under different external pressures (territory, food, predators, *etc.*), the communities of organisms sharing the same ecosystem underwent co-evolution as a survival strategy. As a result, some of their secondary metabolic routes suffered changes over time, gaining the ability to become resistant to their environment by producing certain low molecular weight metabolites. These small molecules are collectively known as natural products.

It has been found that the natural products may act as small ligands for certain macromolecular targets found within the living organisms or, more often, within other living organisms of the same ecosystem.^1^ In this way, the natural products assist the producing organism to sustain the hostilities of the environmental pressures and survive. Interestingly, many human proteins contain structural domains that are very similar to some of the macromolecular targets affected by natural products; therefore, it was not unexpected to find out that the small molecule natural products originally produced with a different aim (reproduction, signaling, communication, defense), can interact with human proteins and modulate different responses.^2^

As a result of the natural selection process, natural products display wide chemical diversity and chemical specificity, which enhances their ability to interact with diverse biorelevant macromolecules, turning them into a unique and rich source of compounds for new drug development.

Empirically, humankind took advantage of the general properties of these natural products, and medicinal plants have been used in virtually all cultures as a source of medicines since the beginning of time.^3^ Furthermore, statistical analysis performed on the new chemical entities reported between 1981 and 2018 confirmed this situation, revealing that 40% of them were derived from natural products or were natural products themselves. This proportion increases to 64% among anticancer drugs and to 75% when only antibiotics are considered.^4^

Since the abundance of natural products in extracts is invariably low and therefore the former are often isolated in tiny amounts, when they show signs of being useful, it is usually found that their natural supply is not enough to satisfy the demand at a reasonable scale, such as that needed for biological testing. At this point, the development of efficient synthetic pathways to guarantee convenient access to these compounds becomes a requirement.

Furthermore, natural products are currently seen not only as potential sources of inspiration for the development of new medicines through structural diversification; they are also highly regarded as potentially suitable probes to explore the interactions within and between cells, as a logical means to understand the inner-workings of the complex molecular machinery that sustains life.^5^ However, since Nature has produced such wonderfully complex molecules that no synthetic chemist could ever dream of, natural products are still a relevant challenge to organic chemists, who advance the frontiers of knowledge by devising new strategies and reagents for their synthesis.

## 2. Known naturally-occurring indoloquinoline alkaloids

Naturally-occurring indoloquinolines are a small family of alkaloids, which have been isolated mainly from *Cryptolepis sanguinolenta* (Lind.) Schlechter, family Asclepiadaceae.^6^ However, other plants such as *Justicia betonica* L. (Acanthaceae),^7^*J. secunda*^8^ and *Sida rhombifolia* L. (Malvaceae)^9^ are also natural sources of alkaloids that exhibit the indoloquinoline motif. These unique natural heterocycles bear both indole and quinoline rings, fused through their pyrrole and pyridine rings.

**Fig. 1:**
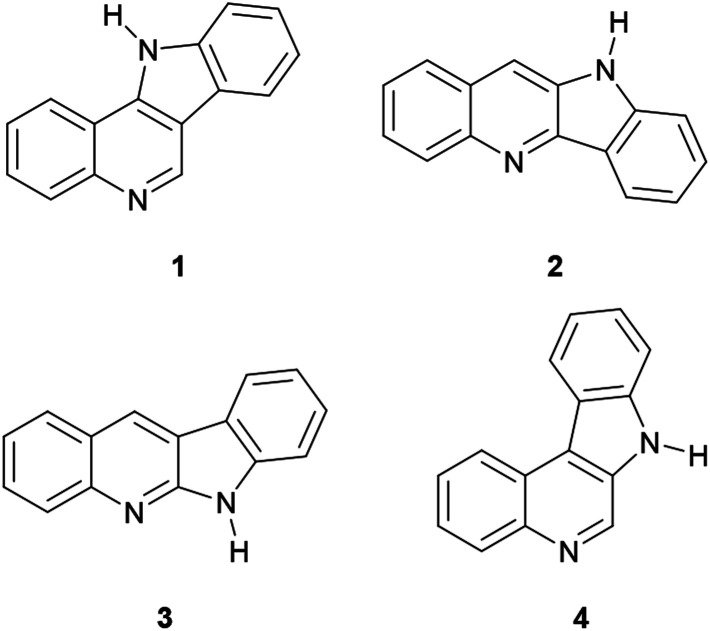
Some isomers of the indoloquinoline skeleton.

Considering this limiting characteristic, only four isomeric ring systems are possible (Fig. 1), namely indolo[3,2-*c*] quinoline (1), indolo[3,2-*b*]quinoline (quinindoline, 2), indolo [2,3-*b*]quinoline (3) and indolo[2,3-*c*]quinoline (4). The cores of natural indoloquinolines correspond to isomers 1–3, and despite the skeleton of 4 being synthesized, there are no natural examples of compounds of class 4.^10^

**Fig. 2:**
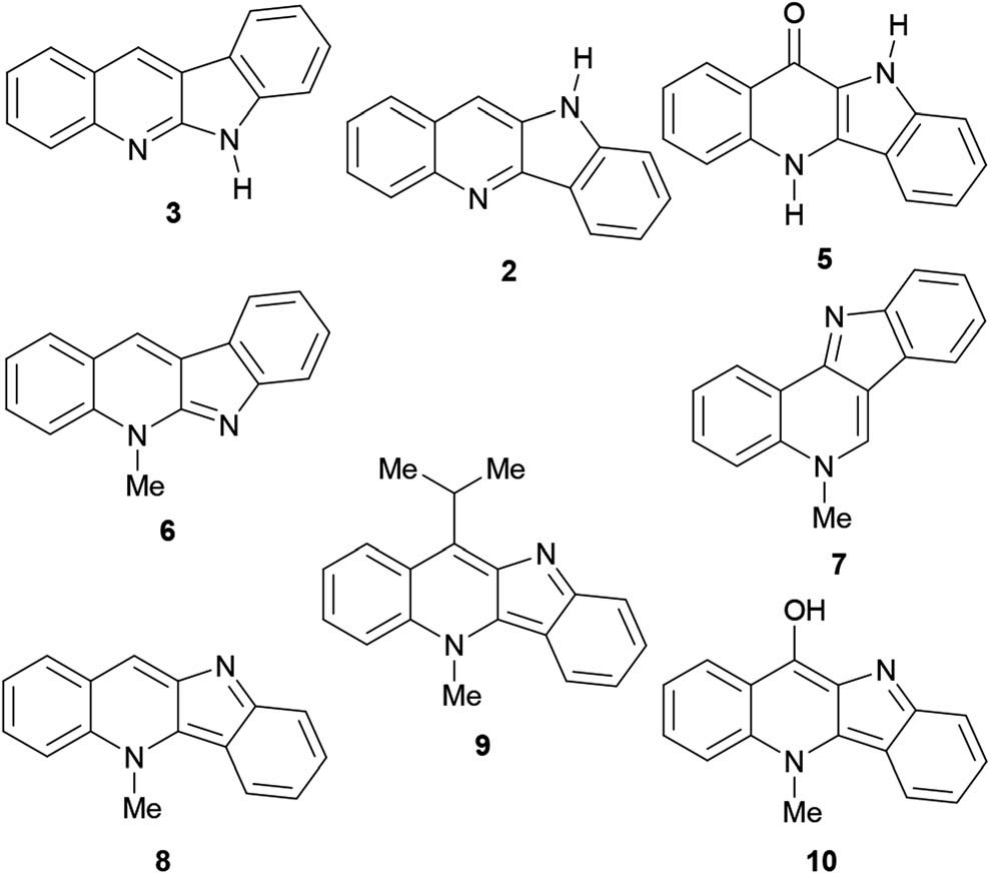
Monomeric naturally-occurring indoloquinolines.

Their main source of indoloquinolines in Nature, *Cryptolepis sanguinolenta*, is a tropical scrambling and twinning shrub, indigenous to West and Central Africa.^11^ The study of the plant began in the early 1950s and cryptolepine was the first alkaloid to be isolated.^12^ To date, over a dozen naturally occurring indoloquinolines have been discovered, including quinindoline (3),^7^ quindoline (2),^13^ quindolinone (5),^14^ neocryptolepine (6),^15^ cryptosanguinolentine (isocryptolepine, 7),^16^ cryptolepine (8),^17^ 11-isopropylcryptolepine (9)^18^ and cryptolepinone (hydroxycryptolepine, 10).^19^ These are monomeric members of this family (Fig. 2), however, several dimeric natural indoloquinolines have also been reported.^19–21^

The plant has long been employed in the dyeing of textiles and leather.^22^ In addition, a decoction of its roots is frequently used in traditional folk medicine, to treat fevers, malaria,^23^ upper respiratory infections and venereal diseases.^24^ The root has been used in the Congo as a bitter stomachic,^24*c*^ and in Nigeria for the therapy of colics, rheumatism, and urogenital infections.^25^ The plant has been demonstrated to possess antibacterial activity and to exert vasodilation.^21*b*^ The pharmacological activities of different indoloquinoline alkaloids, their analogs and derivatives, have been reported in several articles. The most relevant ones are their antitumoral and antiparasitic activities.^26^

## 3. Isocryptolepine: its isolation and properties

Isocryptolepine was first obtained as a degradation product during investigations on the constitution of indigo-yellow.^27^ However, the heterocycle was first isolated from a natural source (*Cryptolepis sanguinolenta*) in 1995 by the Bodo group as a non-crystalline substance, along with cryptolepine and quindoline. Only 5 mg were obtained from 60 g of air-dried roots ground to a fine powder (0.08% w/w). Its chemical structure was determined after exhaustive analysis of its infrared (IR) and nuclear magnetic resonance (NMR) spectroscopic and mass spectrometric (MS) and data.^16^ Interestingly, on the bases of spectral grounds, it has also been conjectured that the previously isolated and partially characterized alkaloid CSA-3 may be identical to isocryptolepine.^13^

Shortly after, the same alkaloid was isolated again by the Tackie group, from the same source in 1996, under the name cryptosanguinolentine.^28^ The natural product, obtained as a yellowish residue (1.7 mg from 3.14 kg of plant roots; 0.5 × 10^−4^% w/w), was isolated along with cryptotackieine (neocryptolepine); due to the scarcity of the material, preparative reverse-phase high-performance liquid chromatography (HPLC) and micro NMR techniques were required to confirm its structure. At a later date, the same group reported a more detailed account of their isolation, with the obtaining of another 15 alkaloids.^21*a*^

The ^1^H, ^13^C and ^15^N NMR spectra of isocryptolepine and its protonated form were assigned as part of a systematic NMR study of four isomeric indoloquinoline alkaloids.^29^ All of the NMR signals were assigned using 2D correlation techniques. Interestingly, in the ^15^N NMR [a gradient selected single-quantum multiple-bond correlation (GSQMBC) experiment], the quinoline-N (N-5) was observed in the usual range at *δ* 135.6 ppm, whereas the indole-N (N-11), which has is imine-like in nature, was found to be significantly deshielded (*δ* 248.1 ppm).

Density functional theory (DFT) calculations of the chemical shielding constants were also performed, allowing a detailed investigation of the effects of protonation and solvation. The calculations were able to reproduce the main experimental trends observed upon protonation, and it was observed that the inclusion of solvent effects in the computations improved the agreement with the experimental ^13^C NMR data.

^1^H NMR data was also recorded in order to determine that the p*K*_a_ of the natural product is 9.8. This result was obtained through a graphical estimation of the inflection point and also by the use of the Henderson–Hasselbalch equation [eqn (1)],^30^ which showed a linear dependence of the chemical shift on the pH of the sample:^31^

1 pH = p*K*_a_ + log[*δ*_max_ − *δ*]/[*δ* − *δ*_min_]

The effect of cyclodextrin (CD) encapsulation on the photophysics of isocryptolepine, was studied using steady state and time-resolved fluorescence spectroscopy.^32^ In the excited state, it was observed that the natural product exists mainly in its zwitterionic form, exhibiting appreciable emission from the π–π* state upon excitation at a specific wavelength.

Due to the presence of the hydrophobic nanocavities of CDs, the existence of zwitterions in the excited state allows a mutual interaction to form dimers, triggered through coulombic interactions. This was evidenced by treating the fluorophores with CDs with different cavity spaces and employing steady state fluorescence measurements. Moreover, the photophysical behavior of the heterocycle was found to be modulated by the nature of the cyclodextrin. In addition, trapped water molecules inside the bigger cavity of γ-CD seemed to quench the fluorescence of the zwitterions.

Isocryptolepine has been demonstrated to show antimicrobial,^33^ anti-trypanosomal,^34^ antifungal,^35^ anti-inflammatory, antimalarial (including β-haematin inhibition),^36^ antitrypanosomal, antileishmanial and cytotoxic activities,^34,36*c*,37^ as well as the ability to interact with DNA,^38^ promote low density lipoprotein uptake in HepG2 cells,^39^ and be useful as a treatment for intestinal disorders. Most of these properties are common to other natural indoloquinolines, which differing in potency and selectivity.

As a result of its profile, during the last 20 years, this natural product became a recurrent target for total synthesis as well as a useful scaffold for the preparation of analogs^40^ and derivatives,^41^ with the aim of improving its activity profile (mainly its potency and selectivity). Furthermore, its attractive structure turned its synthesis into a fertile field for testing the scope and efficiency of new and imaginative C–C and C–N bond forming reactions. Detailed below are the different approaches that have been designed to synthesize isocryptolepine.

## 4. Total syntheses of isocryptolepine

Isocryptolepine is an angularly fused indoloquinoline. As observed in the case of other indoloquinolines, there has been some relevant synthetic work that focused on targeting isocryptolepine before it was found in nature, in 1996. However, most of the synthetic efforts toward the alkaloid have been carried out over the last 20 years, where the natural product has been totally synthesized over 25 times, by different groups around the world; furthermore, some of them can be credited with more than one synthesis.

We have previously classified the synthetic approaches toward the indoloquinoline alkaloids neocryptolepine (6) and quindoline (2) into three main groups, based on the chemical structure of the starting materials involved in the sequence, including: (a) benzenoids, (b) quinolines and (c) indoles.^42^ Accordingly, except for the advances recorded before the isolation of isocryptolepine from *C. sanguinolenta*, the total syntheses of the natural product are classified here to fit into these three categories. In addition, for the sake of clarity, the syntheses are ordered chronologically.

### 4a. Early synthetic studies before the isolation of isocryptolepine from a natural source

The first reported total synthesis of the structure of isocryptolepine was disclosed in 1950 by Kermack and Storey,^43^ who synthesized derivatives of the tetracycle 1 for biological testing, in an attempt to find new antimalarials. These authors employed a modification of the Graebe–Ullmann carbazole synthesis for the preparation of the tetracyclic framework of their prospective compounds and also synthesized isocryptolepine *via* the *N*-methylation of 1.

**Scheme 1:**
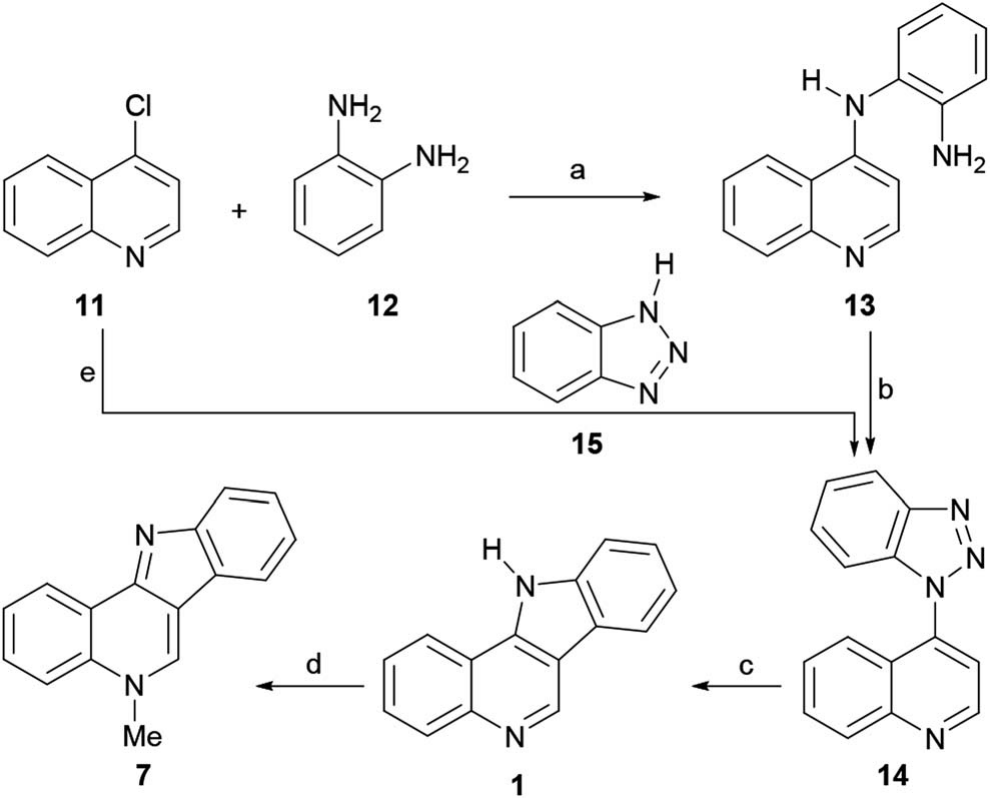
Reagents and conditions: (a) 140 °C, 20–30 mm Hg, 10–20 min (76%); (b) NaNO_2_, 1 N HCl, H_2_O, 5 °C (70%); (c) H_3_PO_4_, heat (77%); (d) MeI, PhNO_2_, 2 h, 100 °C (72%); (e) (1) 15, MW (160 W); (2) pyrophosphoric acid, 160 W, 4–6 min (60% overall).

Thus, 4-chloroquinoline (11) was condensed with *ortho*-phenylenediamine (12) at 140 °C at 20–30 mmHg for 10–20 min, resulting in 4-(2-aminoanilino)quinoline (13), in 76% yield (Scheme 1). After this, 13 was submitted to a Graebe–Ullmann protocol by diazotization with HNO_2_ to afford the benzotriazole derivative 14 in 70% yield. Further heating of 14 with syrupy phosphoric acid, gave 1 in 77% yield, after the loss of nitrogen and rearrangement.

The product was obtained as a violet fluorescent compound, which exhibited increased fluorescence when dissolved in concentrated sulfuric acid. Finally, the tetracycle was heated in a steam bath with excess methyl iodide in nitrobenzene, furnishing the methiodide of 7 in 72% yield. The base of the latter could be freed by treatment with aqueous ammonia, followed by drying of the resulting isocryptolepine monohydrate.

**Scheme 2:**
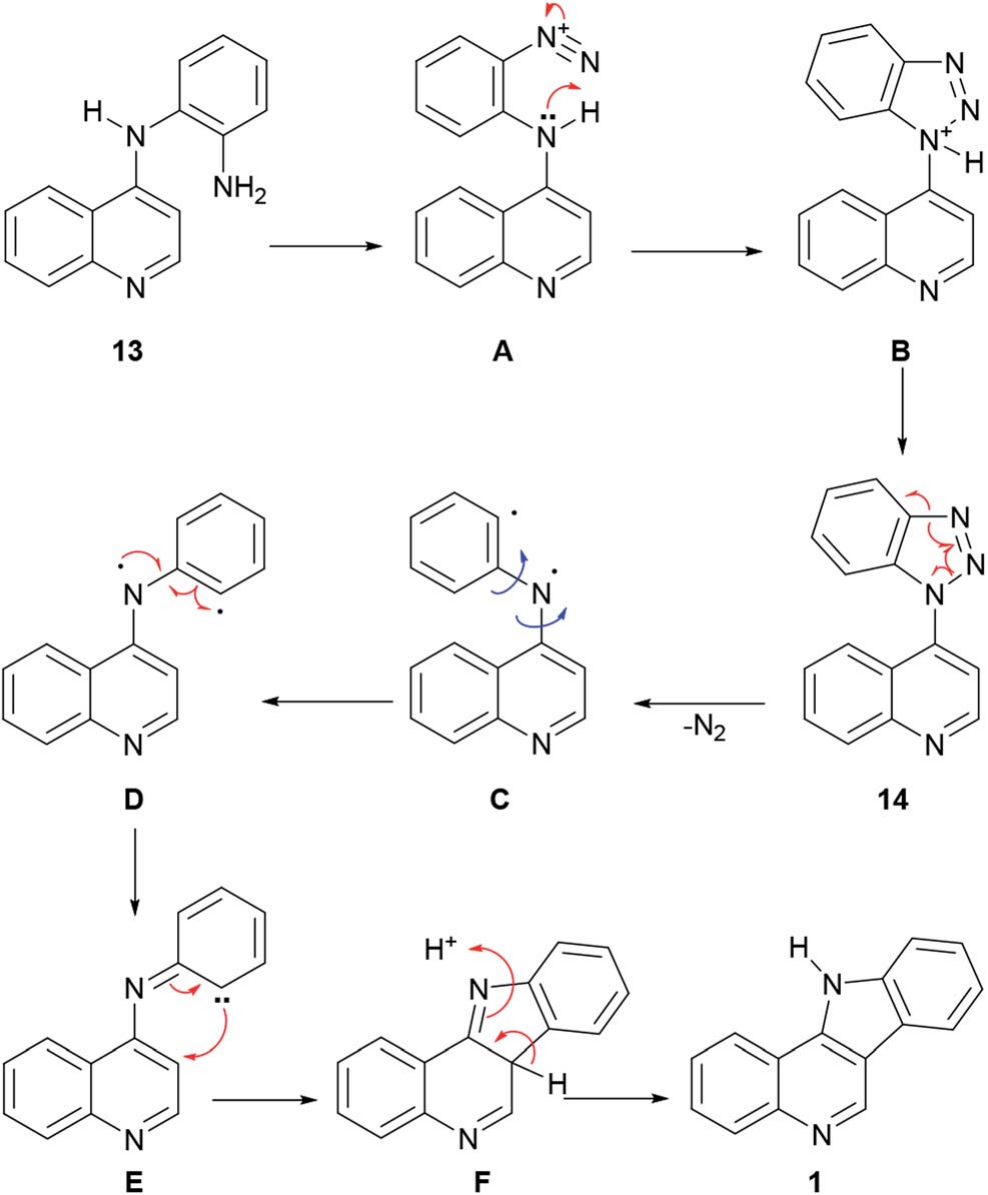
Proposed mechanism of the Graebe–Ullmann synthesis of isocryptolepine.

In the reaction mechanism (Scheme 2), the primary amino group of 13 is diazotized with HNO_2_ to give the diazonium salt intermediate A, which undergoes an intramolecular cyclization to the protonated benzotriazole intermediate B. After deprotonation, benzotriazole 14 loses nitrogen to afford the diradical species C, which in its conformation D undergoes a radical rearrangement to the carbene intermediate E. Then, cyclization takes place to afford intermediate F, which further aromatizes to 1, *via* a [1,3] proton shift.

This contribution complemented the earlier findings by Clemo and Perkin,^44^ which can be regarded as a synthesis of an advanced intermediate of the natural product. These authors demonstrated that upon heating with aqueous sulfuric acid, the phenylhydrazone of 1,2,3,4-tetrahydroquinolin-4-one underwent a one-pot Fischer indolization and subsequent dehydrogenation to give 1. Employing the same approach but starting with the phenylhydrazone of *N*-methyl-1,2,3,4-tetrahydroquinolin-4-one (obtained from *N*-methylaniline in 15% yield) Braunholtz and Mann also synthesized isocryptolepine in 1955 in an unspecified yield.^45^

**Scheme 3:**
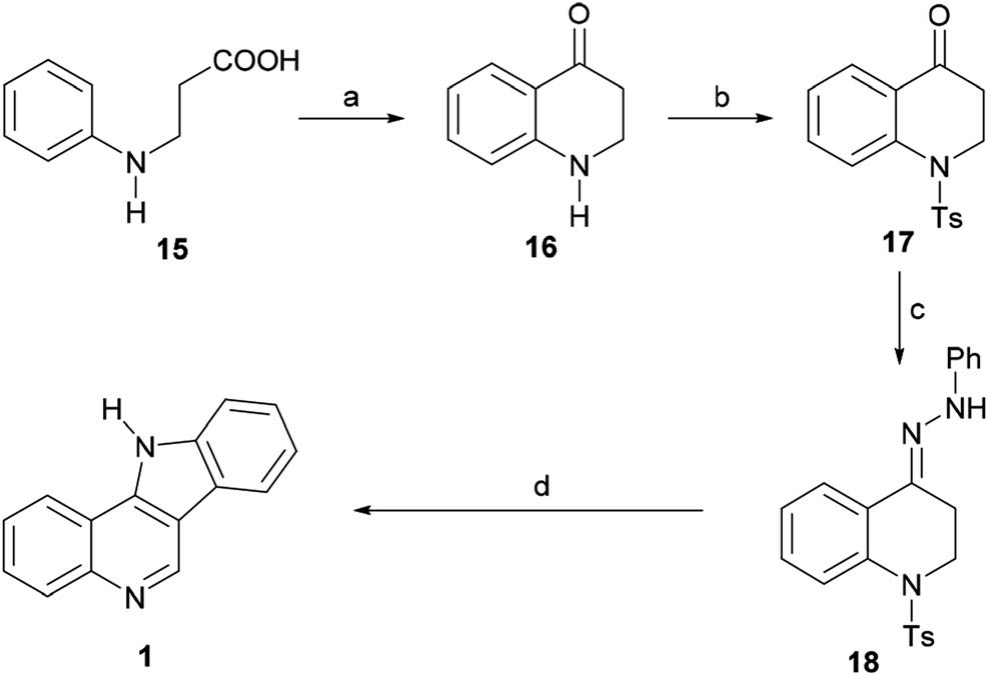
Reagents and conditions: (a) PPA, 130 °C, 20 min (68%); (b) TsCl, Et_3_N, CH_2_Cl_2_; (c) PhNHNH_2_, EtOH, AcOH (cat), 80 °C, 90 min, 74%; (d) AcOH, H_2_SO_4_, 10 min, Δ, 50%.

In 1965, Roussel *et al.* reported an alternative approach toward 1 (Scheme 3) by way of the 1,2,3,4-tetrahydroquinolin-4-one 16.^46^ The ketone was best prepared in 68% yield by direct cyclization of β-*N*-phenylalanine (15, obtained in turn from aniline and methyl acrylate) with PPA at 130 °C for 20 min. However, 16 could also be accessed in three steps and in substantially the same yield *via* the aza-Michael addition of aniline to methyl acrylate, followed by ester hydrolysis and cyclization.

The latter was converted into the tosylamide 17 and further indolized in 50% overall yield by first heating it for 90 min at 80 °C with phenylhydrazine in EtOH, to give the hydrazone 18, and then heating it for 10 min in AcOH, under H_2_SO_4_ promotion. The cyclization stage was accompanied by both detosylation and dehydrogenation toward 1.

**Scheme 4:**
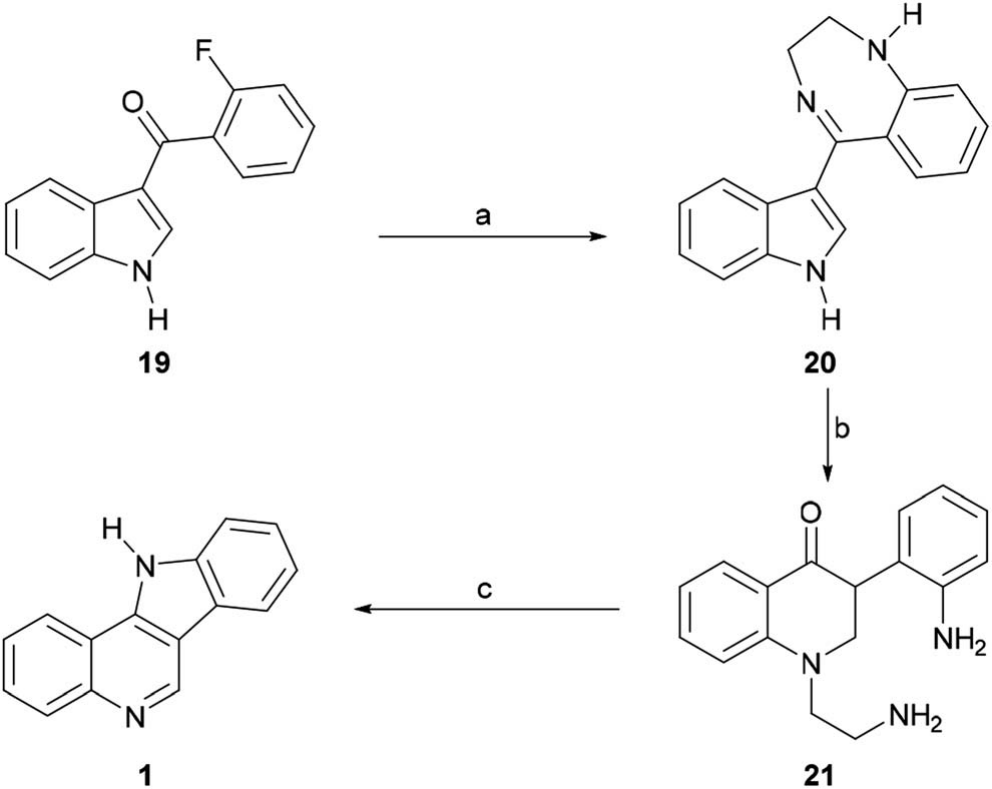
Reagents and conditions: (a) H_2_NCH_2_CH_2_NH_2_, pyridine, reflux, 20 h (54%); (b) 2 N H_2_SO_4_, EtOH, reflux, 72 h (70%); (c) Ph_2_O, reflux (low yield).

In 1968, García *et al.* disclosed an additional alternative process of synthesizing [3,2-*c*]indoloquinoline (1).^47^ These authors prepared 3-(2-fluorobenzoyl)indole (19) *via* the acylation of indolylmagnesium bromide with *o*-fluorobenzoyl chloride (Scheme 4) and found that the subsequent treatment of the latter with ethylenediamine in refluxing pyridine gave the substituted aminobenzoylindole imine derivative 20 in 54% yield *via* the nucleophilic exchange of fluorine for an amine.^48^ Upon acid treatment, 20 underwent rearrangement to afford a mixture of the quinolone 21 and the indoloquinoline 1. However, heating the quinolone in diphenyl ether under reflux gave the fused heterocycle 1 as the sole product, apparently in very low yield.

In 1993, the Alvarez-Builla group improved and simplified the original sequence of Kermack and Storey,^38*a*,49^ by reporting a one-pot Graebe–Ullmann approach to the tetracyclic precursor 1 of the natural product, as a surrogate for the traditional Fischer indolization approach (Scheme 1). The process, which was later employed by Murray *et al.* for the preparation of analogs for biological evaluation as antimalarials,^36*d*^ could be carried out in an open vessel using a domestic microwave oven.

The strategy involved two irradiation stages; first, a solventless mixture of 11 and 15 was irradiated at 160 W to obtain the benzotriazole derivative 14 in 90% yield; then, without purification of the benzotriazole, a second irradiation process was performed at 160 W in the presence of pyrophosphoric acid to produce the tetracycle 1 in 60% yield after 4–6 min. Interestingly, it was observed that the attained yield under these conditions was similar to that obtained by applying conventional heating. It was also concluded that irradiation of a solventless mixture of the pure reactants was more efficient than using their supported forms, over silica gel or montmorillonite.

### 4b. Syntheses from benzenoids

#### Synthesis by Novikov *et al.*

**Scheme 5:**
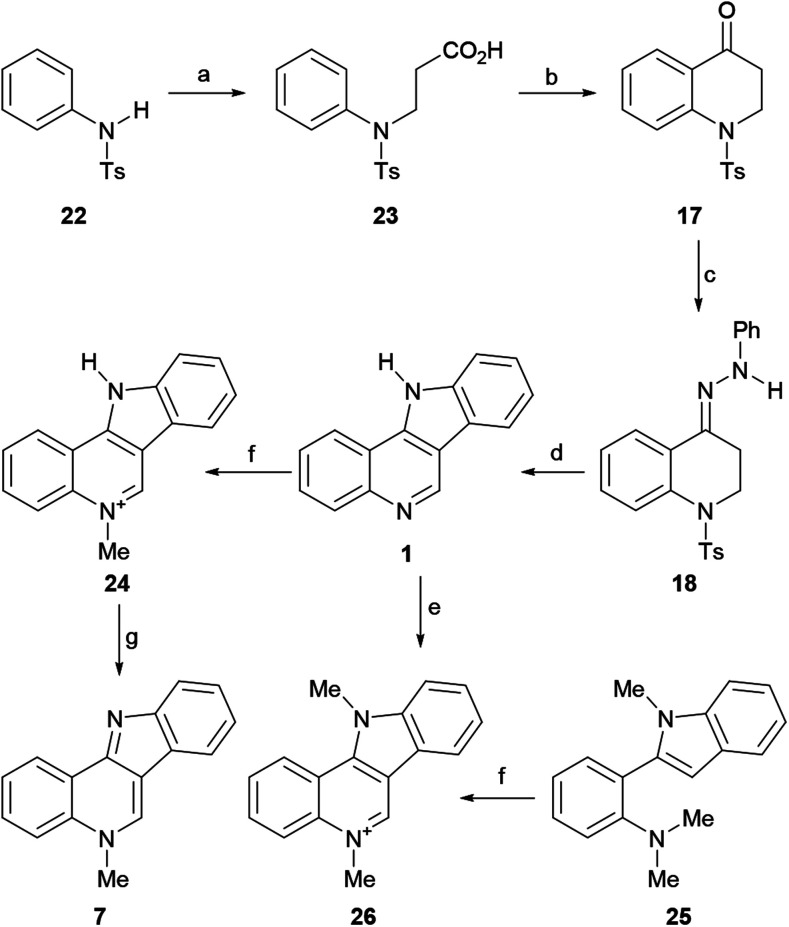
Reagents and conditions: (a) ClCH_2_CO_2_H, NaOH (69%); (b) PPA, 130 °C, 10 min (96%); (c) PhNHNH_2_, AcOH (cat.), EtOH, 80 °C, 1.5 h (87%); (d) H_2_SO_4_, AcOH, 100 °C, 10 min (56%); (e) MeI, K_2_CO_3_, Me_2_CO, reflux, 3 h (82%); (f) MeI, PhMe, ∼20 °C (64%); (g) K_2_CO_3_, H_2_O, ∼20 °C (93%); (f) I_2_, TBHP, CHCl_3_, rt, 14 h (75%).

The first total synthesis of isocryptolepine after its isolation as a natural product was carried out starting from simple benzenoid compounds, reported in 1996 by the Novikov group (Scheme 5).^50^ The synthetic pathway, which was reported in few details, followed the indolization strategy originally devised by Clemo and Perkin.^44^

The final product was obtained from *N*-tosylaniline (22) *via* a six-step sequence in an overall yield of 19.2%. To that end, the authors performed the *N*-alkylation of 22 with β-chloropropionic acid^51^ to obtain the sulfonamide 23 in 69% yield and cyclized the latter by heating at 130 °C with polyphosphoric acid,^52^ to access the required intermediate carbonyl derivative 17 in 96% yield.

In turn, the 1,2,3,4-tetrahydroquinolin-4-one 17 was exposed to phenylhydrazine under AcOH catalysis, furnishing 87% yield of the intermediate hydrazone 18. Subsequently, this compound was subjected to indolization with H_2_SO_4_ in acetic acid at 100 °C, to give 1 in 56% yield. Exposure of the tetracycle 1 to MeI afforded the mono-methiodide 24 in 64% yield. Final basic treatment to free the base gave isocryptolepine (7) in 93% yield. Interestingly, in the presence of K_2_CO_3_, the bis-*N*-methylated isocryptolepinium derivative 26 was obtained in 82% yield. More recently, 26 was accessed by Volvoikar and Tilve in 75% yield from 25, employing a cross-dehydrogenative coupling reaction with TBHP and I_2_.^53^

**Scheme 6:**
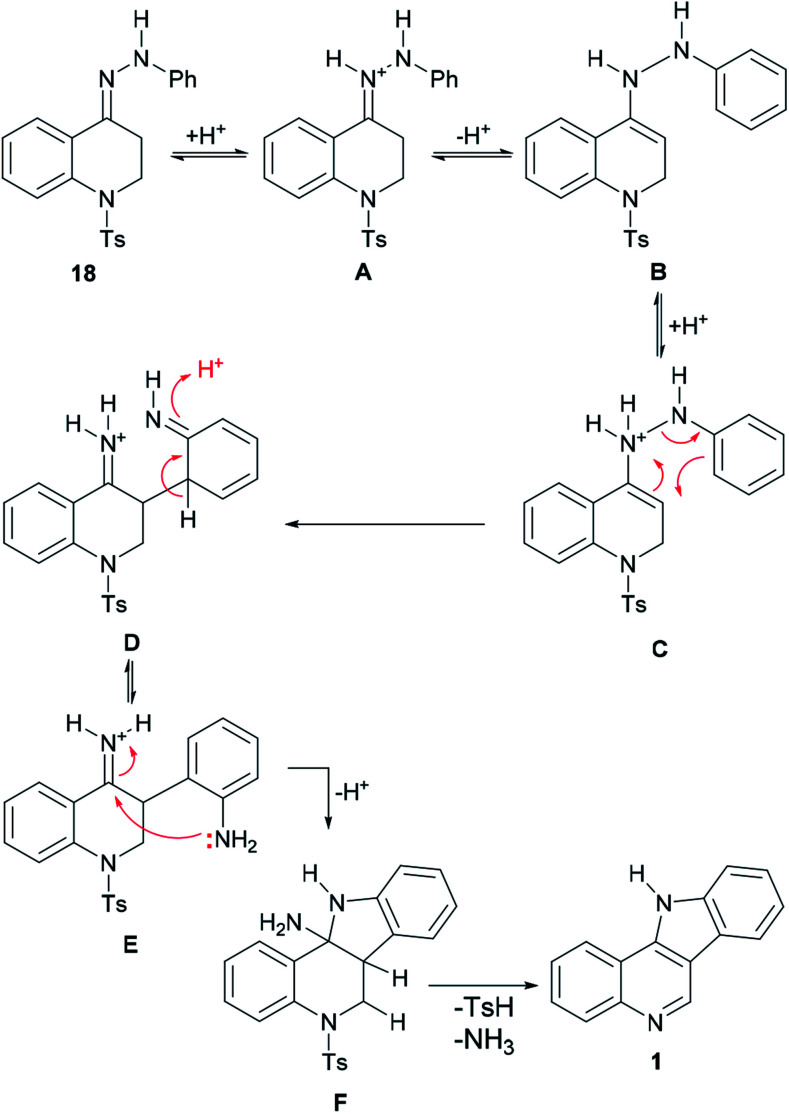
Mechanism of the indolization of hydrazone 18 toward indoloquinoline 1.

The mechanism of the transformation of the hydrazone intermediate 18 into the tetracycle 1 is depicted in Scheme 6. It is proposed that the protonation of 18 to afford A would give B after tautomerization. In turn, the latter could become protonated (C) and rearrange to D followed by rearomatization to give the anilino-iminium ion E. Nucleophilic attack of the aniline of the iminium moiety would afford intermediate F, which could then provide 1 after losing ammonia and then toluenesulfinic acid, driven by the possibility of aromatization.

#### Synthesis by Molina *et al.*

The Molina group, which has been active in the synthesis of indoloquinolines and related heterocycles through the use of intramolecular aza-Wittig reactions, presented in 1999 their total syntheses of both cryptotackieine (neocryptolepine) and isocryptolepine from a common key intermediate, *via* a selective indolization process.^54^

**Scheme 7:**
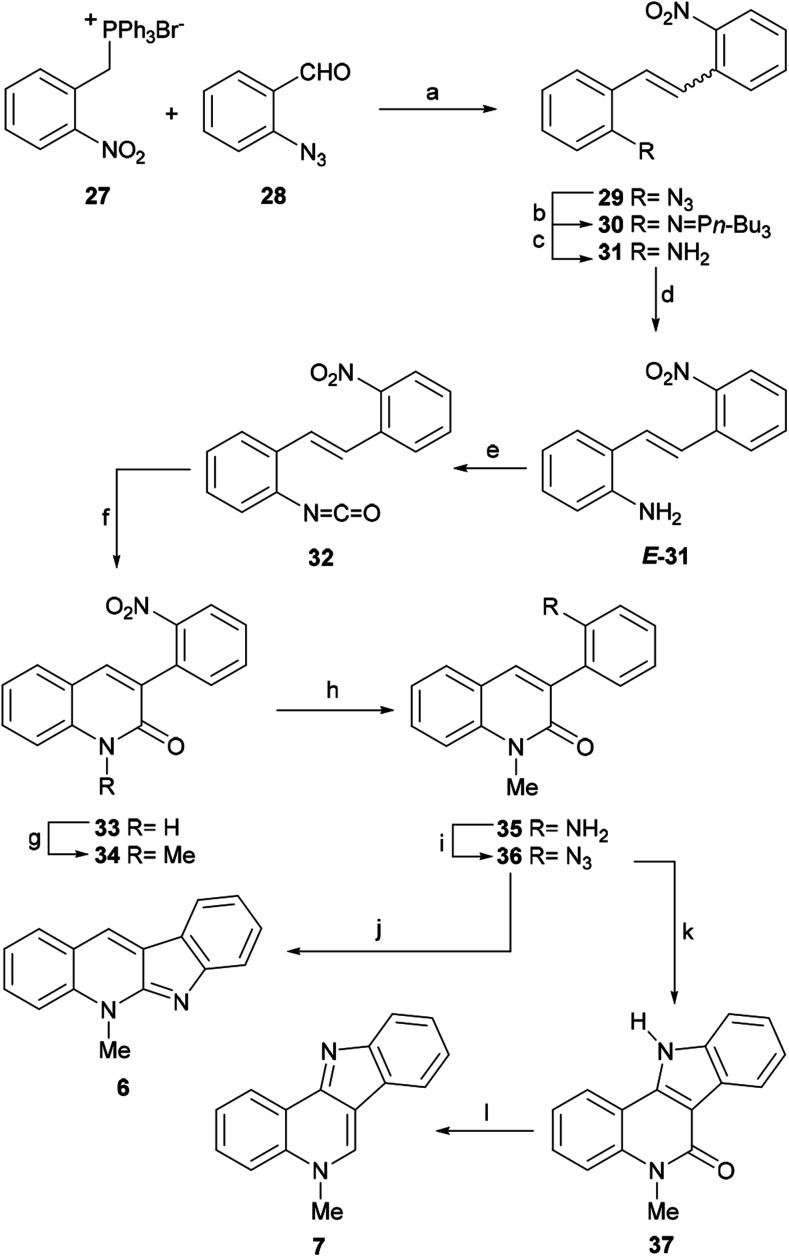
Reagents and conditions: (a) K_2_CO_3_, dibenzo-18-crown-6, CH_2_Cl_2_, rt (85%, *Z*/*E* = 4 : 1); (b) *n*-Bu_3_P, CH_2_Cl_2_, rt; (c) THF–H_2_O, rt (84%, *Z*/*E* = 7 : 1); (d) PhSH, AIBN, PhH, reflux (92%); (e) triphosgene, CH_2_Cl_2_, Et_3_N, 0 °C → rt, 1 h; (f) PhNO_2_, MW, 150 °C, 12 min (80% overall); (g) CH_3_I, DMF, 60 °C (82%); (h) H_2_, Pd/C, EtOH, rt (91%); (i) (1) NaNO_2_, H_2_SO_4_; (2) NaN_3_, H_2_O (85%); (j) (1) Me_3_P, PhNO_2_, rt, 45 min; (2) MW, 180 °C, 30 min (40% overall); (k) *o*-xylene, 150 °C (82%); (l) Red-Al, PhMe, reflux, 32 h (90%).

In their rather long synthetic sequence, the phosphonium salt 27 was condensed with the azidobenzaldehyde 28 employing K_2_CO_3_ and dibenzo-18-crown-6, to give the stilbene derivative 29 in 85% yield as a 4 : 1 mixture of *Z* : *E* isomers (Scheme 7). Next, the Staudinger reaction of the azide 29 with *n*-Bu_3_P provided the iminophosphorane 30 which was hydrolyzed to the aniline 31 in 84% overall yield, as a mixture of isomers (*Z* : *E* = 7 : 1). Subsequently, the catalytic isomerization of the double bond with the thiophenol/AIBN reagent system afforded *E*-aminostilbene 31 in 92% yield. This compound contains the nitrogen atoms required to install both heterocyclic rings.

Treatment of *E*-31 with triphosgene and further microwave-promoted cyclization of the resulting isocyanate 32 furnished the quinolin-2-one derivative 33 in 80% overall yield.^55^ The latter was next transformed into the common intermediate 36 by means of a three-step sequence involving *N*-methylation to give 34 (82% yield) followed by Pd/C-mediated catalytic hydrogenation to afford 35 in 91% yield and final one-pot treatment of the amine with HNO_2_, and subsequent reaction of the resulting diazo intermediate with NaN_3_ to provide the azide derivative 36 in 85% yield.

The indolization of the azide 36 toward the framework of isocryptolepine was performed by refluxing the azide in *ortho*-xylene, which afforded 37 (isocryptolepinone) in 82% yield. Interestingly, 37 was previously prepared from *N*-methyl-4-azido-3-phenylquinolin-2-one, by refluxing in dimethylformamide (DMF) for 2 h (72% yield) through quite a similar nitrene-based reaction, and in 61% yield *via* irradiation with a low pressure mercury lamp at room temperature for 4 h.^56^ More recently, the Sagar group reported the synthesis of the lactam 37 in 68% yield *via* the reductive cyclization of 34 under MoO_2_Cl_2_(DMF)_2_ catalysis.^57^

Final reduction of the carbonyl moiety with Red-Al in toluene at reflux gave isocryptolepine (7) in 90% yield. Interestingly, treatment of 36 with the highly reactive Me_3_P gave an intermediate iminophosphorane, which upon microwave irradiation in nitrobenzene at 150–180 °C for 30 min furnished cryptotackieine (6) in 40% yield *via* an aza-Wittig type reaction.^58^

#### Synthesis by Kundu *et al.*

In 2009, the Kundu group reported an alternative synthesis of isocryptolepine.

Unlike most of the previous strategies, which relied predominantly on the formation of the indole by ring closure to culminate the synthesis, their approach was characterized by the formation of the quinoline ring in the final step.^59^

**Scheme 8:**
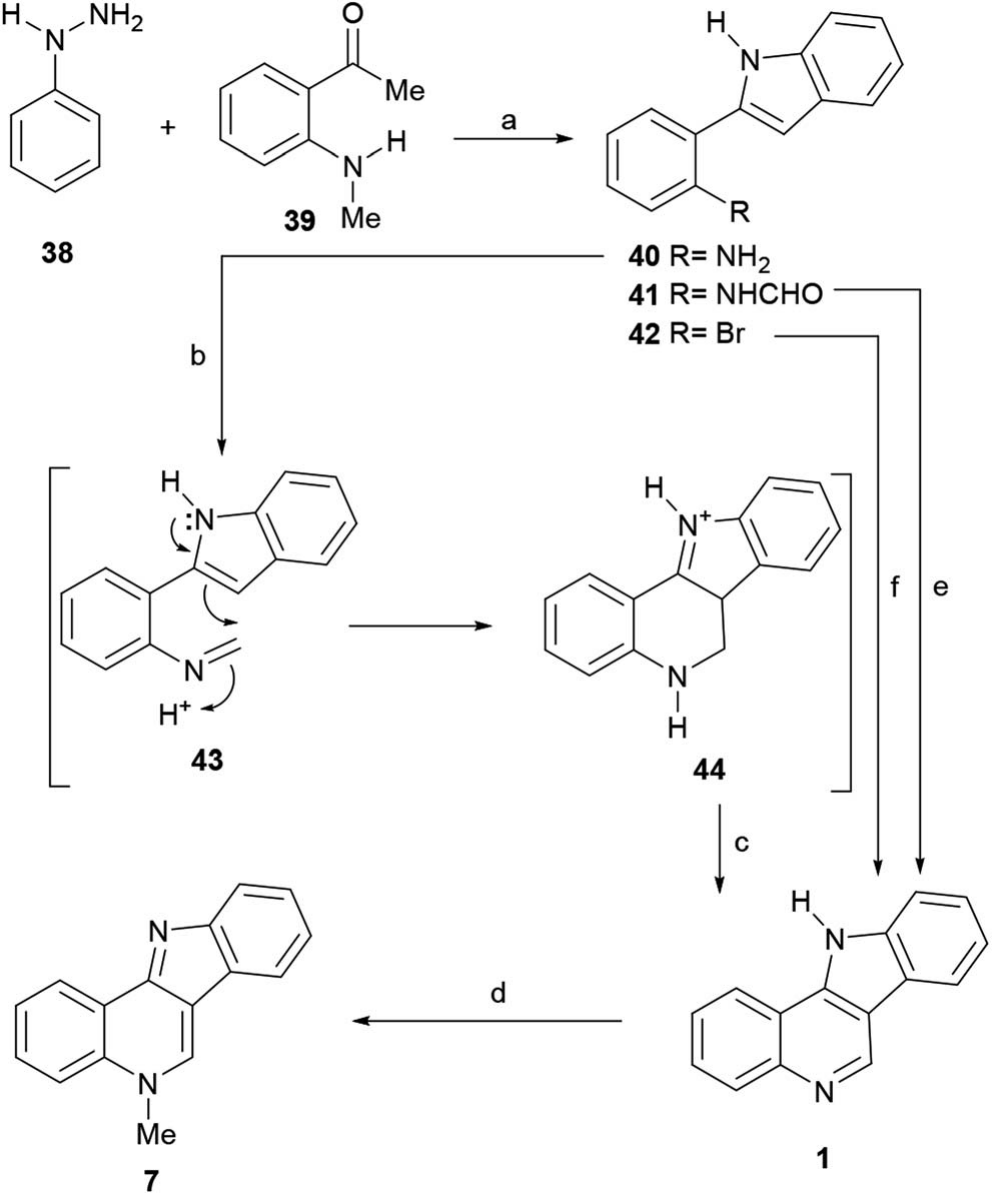
Reagents and conditions: (a) (1) AcOH (5 mol%), EtOH, 6 h; (2) PPA, 100 °C, 10 min; (b) (CH_2_O)_*n*_, TFA, MeCN, 80 °C (sealed tube), 2 h; (c) air [O] (86% overall); (d) MeI, PhMe, reflux, 2 h; (e) Tf_2_O, Ph_3_PO, r.t., 15 min (75%), (f) (1) HCOH, CuI, l-proline, NH_3_·H_2_O, K_2_CO_3_, DMSO 100 °C, 12 h; (2) HCl, air, 120 °C, 8 h (32%).

The sequence was based on Kundu's modified version of the Pictet–Spengler cyclization.^60^ To find the correct conditions, they first put in place a Fischer indole synthesis between phenylhydrazine (38) and 2-methylaminoacetophenone (39)^61^ to afford the intermediate 40 (Scheme 8).^62^

The latter was then subjected to a Pictet–Spengler reaction with paraformaldehyde in the presence of TFA, to directly furnish the indoloquinoline 44. Presumably 44 was formed through the intermediacy of imine 43. In turn, 44 underwent spontaneous aerobic oxidation to afford 1 in 86% yield.^63^ Then, the tetracycle was subjected to selective *N*-methylation, by treatment with MeI in refluxing toluene to furnish isocryptolepine.

Interestingly, when exposed to Hendrickson's reagent (Tf_2_O-PPh_3_O), the *N*-formyl derivative 41 gave 1 in 75% yield, through a regioselective 6-*endo*-cyclization.^64^ The precursor 41 was elaborated efficiently *via* a gold(iii)-catalyzed 5-*endo-dig* cyclization.^65^ On the other hand, a regioselective copper-catalyzed one-pot cascade reaction of bromoarene 42 with formaldehyde and aqueous ammonia in DMSO has been developed, which affords 1 in 32% yield.^66^

#### First synthesis by Kraus *et al.*

Kraus and coworkers developed a flexible and high yield synthesis of indoles based on the electrocyclic ring closure of the anion of a Schiff base derived from the reaction of the commercial 2-aminoaryl-benzylphosphonium salt 45 with an aromatic aldehyde.^67^ In 2010, they applied their strategy to develop a formal total synthesis of isocryptolepine, starting with 2-nitrobenzaldehyde (46).^68^

**Scheme 9:**
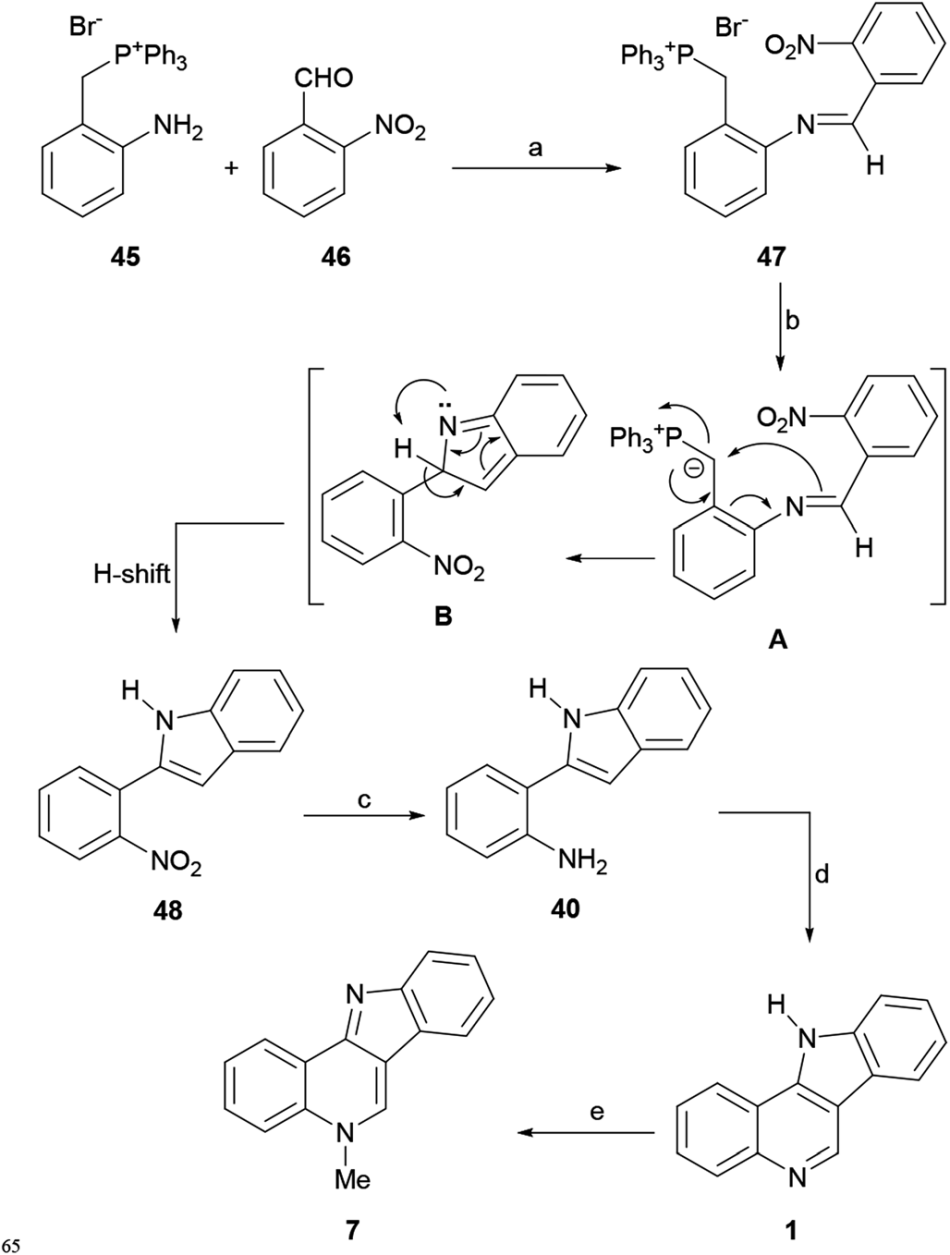
Reagents and conditions: (a) (1) AcOH, MeOH, MW (80 °C), 10 min (72%); (b) K^*t*^BuO, THF, 25 °C, 1 h; (c) Fe^0^, HCl, EtOH (90%); (d) (CH_2_O)_*n*_, TFA, MeCN, 80 °C (81%); (e) MeI, PhMe (91%).

Thus, the required imine 47 was synthesized by a reaction between 45 and 46 under AcOH catalysis, employing microwave irradiation at 80 °C on a methanolic solution of the reactants (Scheme 9). The reaction of the as-formed nitro-imine 47 with K^*t*^BuO in THF at 0 °C gave the indole 48 in 72% overall yield, presumably through the anionic species A, which may lose PPh_3_ after undergoing cyclization to the intermediate B, which in turn undergoes a final H-shift and aromatization.

Reduction of the nitro group with elemental iron (Fe^0^) and HCl in EtOH to aniline 40 and further formylation of the indole moiety with paraformaldehyde and TFA with subsequent *in situ* cyclization completed the tetracycle 1 in 82% yield. Finally, according to Agarwal *et al.*,^59^ the methylation of 1 with MeI in toluene gave 7 in 91% yield.

#### Second synthesis by Kraus *et al.*

**Scheme 10:**
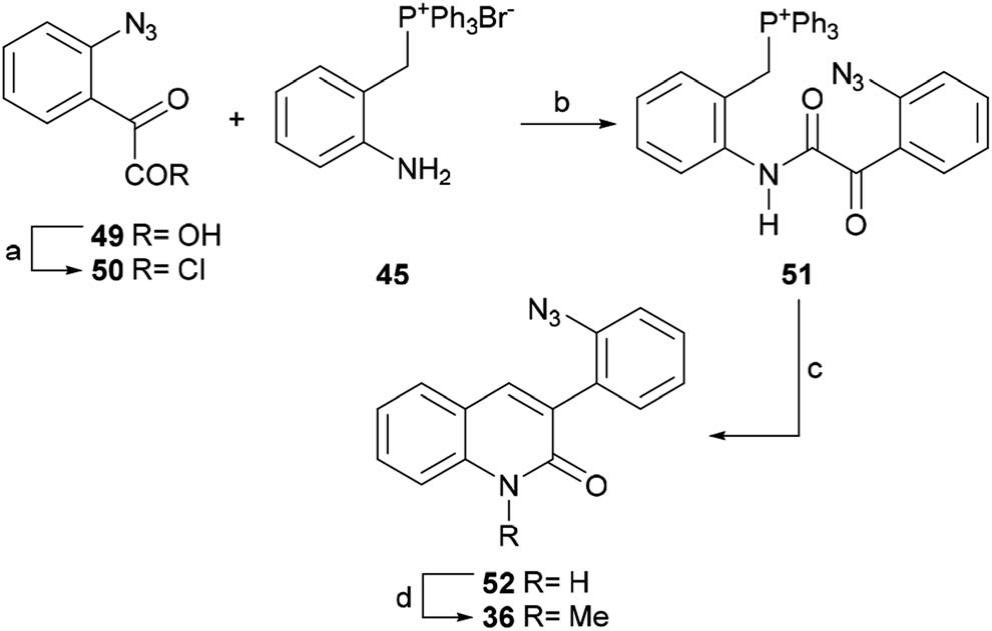
Reagents and conditions: (a) SOCl_2_, PhH, reflux, 1 h or (COCl)_2_, CH_2_Cl_2_, rt, 3 h; (b) CH_2_Cl_2_, rt, 12 h; (c) K^*t*^BuO, THF, rt, 5 h (62% overall); (d) MeI, K_2_CO_3_, DMF, 60 °C, 8 h (98%).

In 2010, the same group reported another application of the commercial 2-aminoaryl-benzylphosphonium salt 45 as an alternative formal total synthesis of isocryptolepine.^69^ To that end (Scheme 10), they started with the known carboxylic acid 49, easily available from isatin in 92% yield,^70^ and prepared the acid chloride 50 by reaction with thionyl chloride or oxalyl chloride.

The product was coupled with the phosphonium bromide 45 and the resulting amide 51 was treated with potassium *tert*-butoxide at room temperature, where it underwent an intramolecular Wittig reaction to afford lactam 52 in 62% overall yield from 49.

The *N*-methylation of 52 with methyl iodide in the presence of K_2_CO_3_ in DMF gave 36 in 98% yield. This lactam is a key intermediate in the total synthesis of isocryptolepine by Molina.^54*b*^ The latter group invested in nine steps to arrive at the intermediate 36, in 22% overall yield, whereas this shortcut advantageously accomplished the same purpose in 60% yield over 4 steps in only two steps.

#### Synthesis by Butin *et al.*

**Scheme 11:**
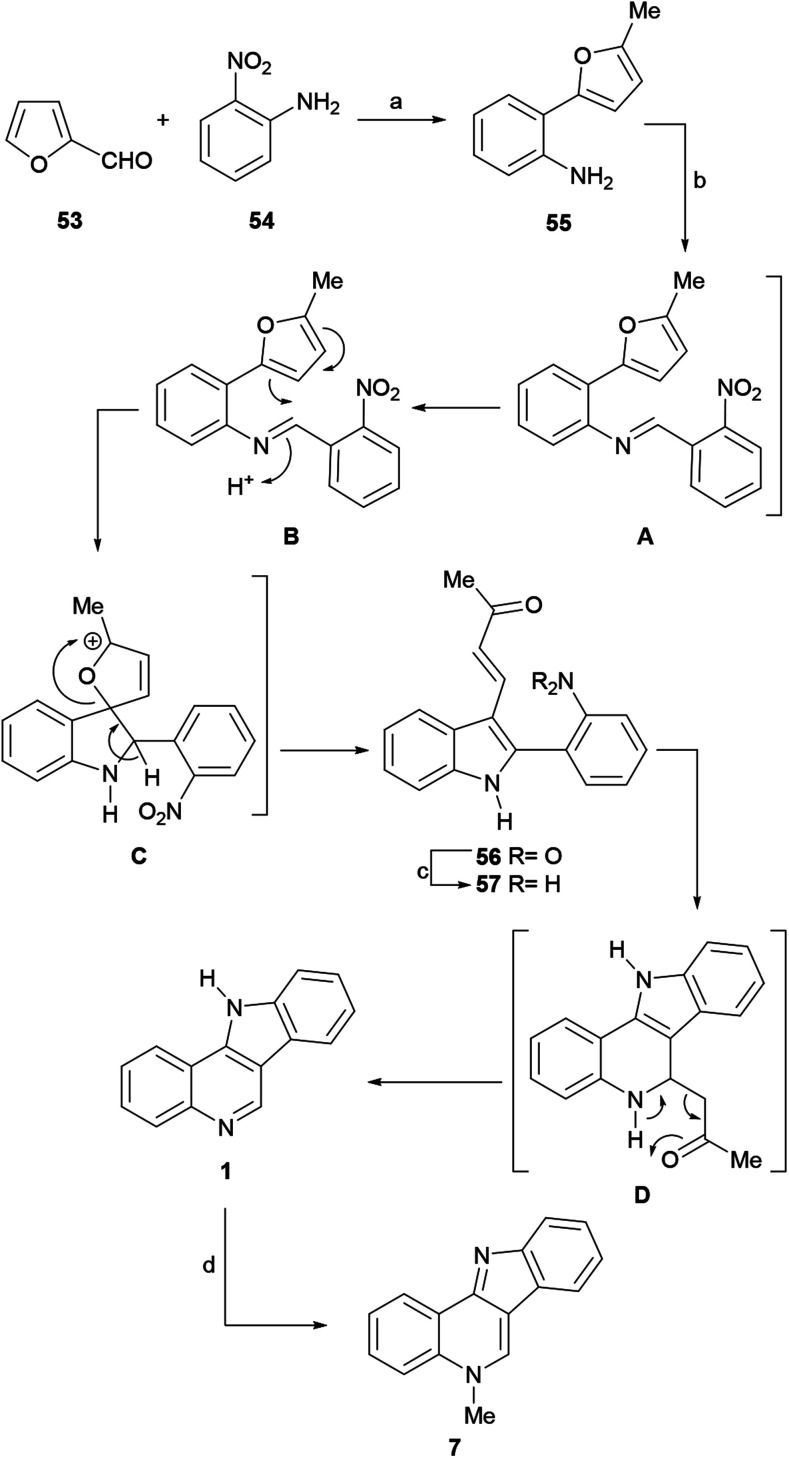
Reagents and conditions: (a) (1) HCl, 30 min, 80 °C; (2) NaNO_2_, 0 °C, 40 min; (3) furfural, CuCl_2_, H_2_O (53%); (4) NaBH_4_, AlCl_3_, THF, 0 °C, 20 min; then reflux, 2 h (67%); (5) RANEY® nickel, NH_2_NH_2_, EtOH, reflux, 2 h (76%); (b) 2-NO_2_C_6_H_4_CHO, HCl, AcOH, 30–35 °C, 1.5 h (58%); (c) Fe^0^, AcOH, reflux, 5 min (86%); (d) (1) MeI, PhNO_2_; (2) NH_4_OH, H_2_O (86%).

The Butin group developed a procedure for the transformation of furfural (53) into 2-furylaniline (55). This was performed *via* a coupling reaction between 53 and the diazo-derivative of 2-nitroaniline (54), followed by sequential reductive deoxygenation of the nitro and formyl moieties.^71^ The authors demonstrated that compound 55 can be further transformed into 3-(2-acylvinyl)-2-(hetero) arylindole (56) through an unusual furan-to-indole recyclization process (Scheme 11).^72^ The substrate scope of this imaginative approach to the synthesis of more complex indole derivatives was demonstrated by the authors through the total synthesis of isocryptolepine (7) and the preparation of differently decorated analogs of the natural product.^73^

The reaction of 55 with 2-nitrobenzaldehyde afforded the expected indole derivative 56, but in comparative lower yield (58%) than for other aldehydes. This was explained as being the result of a combination of unfavorable steric and electronic effects of the *ortho*-nitro moiety, which affected the iminium ion attack on the furan ring.

A possible reaction mechanism includes a first step, where the Schiff base A is formed by the condensation of 55 with the aldehyde. Then, protonation of this intermediate (B) should drive the ring opening of the furan motif and afford the oxaaza-spirocyclic intermediate C, which after undergoing ring opening of the dihydrofuran moiety triggered by aromatization of the nitrogen ring, affords the 2-substituted indole 56.

In the next stage, the reduction of the nitro group, various reagent systems were studied (Zn/AcOH, Zn/NaOH, RANEY® nickel, Fe/AcOH), with the finding that Fe in AcOH directly affords the indoloquinoline 1 in 86% yield. The formation of 1 could result from the reduction of the nitro group to the corresponding aniline 57, followed by an intramolecular Michael addition to give intermediate D,^74^ and aromatization of the pyridine ring of the latter *via* the rather unusual elimination of acetone.^75^ For the *N*-methylation stage, the best result (86% yield) was obtained using the Kermack–Storey procedure, employing MeI in nitrobenzene.^43^

#### Synthesis by Bogányi and Kámán

In 2013, Bogányi and Kámán synthesized different indoloquinoline skeletons from bromoiodoquinolines *via* the consecutive application of two Pd-catalyzed reactions, namely a regioselective Buchwald–Hartwig amination and a subsequent Heck-type biaryl coupling.

**Scheme 12:**
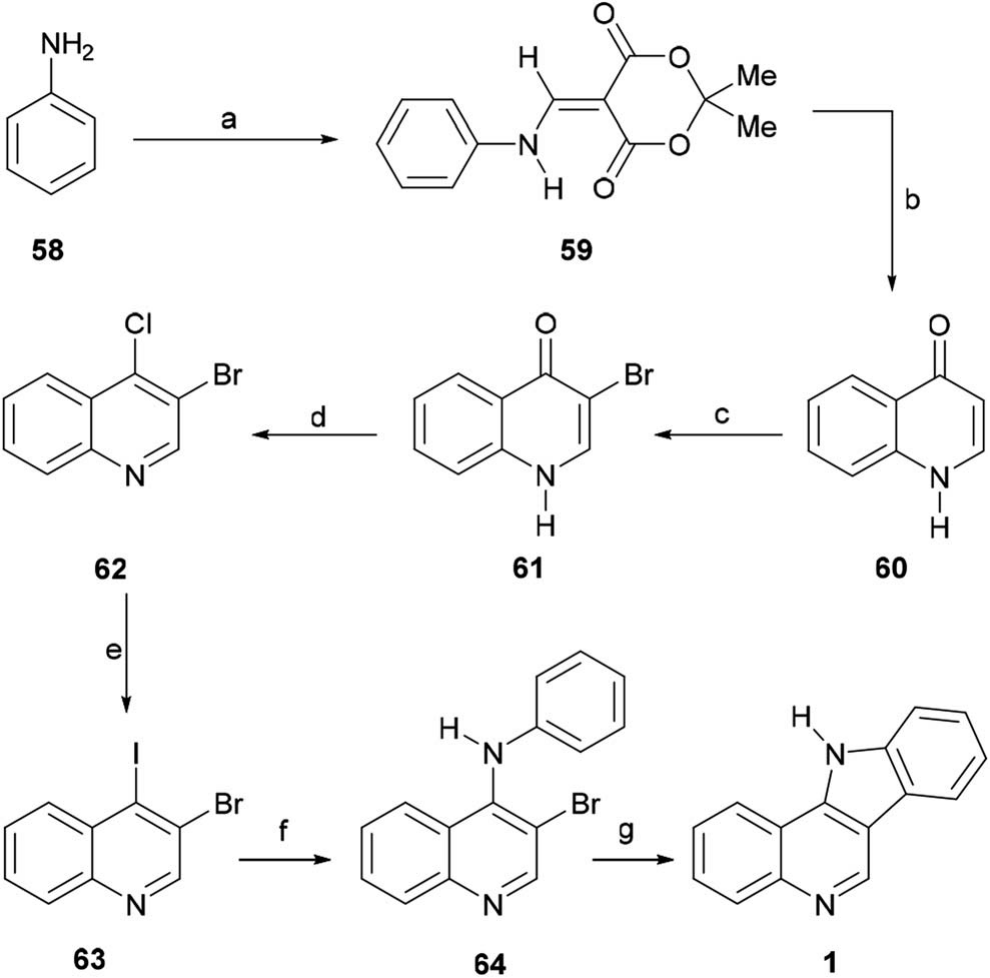
Reagents and conditions: (a) Meldrum's acid, HC(OMe)_3_, reflux (77%); (b) Ph_2_O, 250 °C (92%); (c) Br_2_, AcOH, reflux (99%); (d) POCl_3_, MeCN, reflux (93%); (e) NaI, MeCN, reflux (55%); (f) PhNH_2_, Pd(OAc)_2_, Xantphos, Cs_2_CO_3_, PhMe, MW (120 °C), 1.5 h (95%); (g) PdCl_2_(PPh_3_)_2_, NaOAc, DMA, MW (150 °C), 1 h (64%).

Their contribution included the preparation of the tetracycle 1, which constitutes a formal total synthesis of isocryptolepine.^76^ For this purpose, the authors specifically prepared the 3,4-dihaloquinoline 63 (Scheme 12).

The key 1*H*-quinolin-4-one intermediate 60 was obtained in 77% yield, *via* the condensation of the aniline 58 with Meldrum's acid in the presence of trimethyl orthoformate to afford 59, followed by cyclative thermolysis of the latter in diphenyl ether.^77^ The direct halogenation of 60 with bromine in AcOH^78^ gave the expected 3-bromo-1*H*-quinolin-4-one 61 in 99% yield, whilst treatment of the latter with POCl_3_ afforded 3-bromo-4-chloroquinoline (62) in93% yield. Final installation of an iodide motif was accomplished *via* a Finkelstein-like displacement of the chloride with NaI in MeCN, affording the desired heterocycle 63 in 55% yield.^79^

Next, the dihaloquinoline 63 was coupled with aniline in toluene, employing palladium catalysis, with Xantphos as a ligand and Cs_2_CO_3_ as a base, under microwave irradiation at 120 °C for 90 min, to selectively afford 64 in 95% yield. Final cyclization of 64 to the indoloquinoline motif 1 was performed in 64% yield, through an intramolecular Heck-type reaction in DMA, under microwave irradiation (1 h at 150 °C), using PdCl_2_(PPh_3_)_2_ as a catalyst and NaOAc as a base.^80^

The Maes group prepared analogous intermediates through a different synthetic sequence and developed several syntheses of various indoloquinolines. An interesting advantage of the Kámán synthesis over the previous reports by Maes *et al.* on the tandem palladium-catalyzed approaches to indoloquinolines is that in the latter cases, small amounts of the undesired regioisomer were detected after the ring closure.^10*a*,80^

#### Synthesis by Lu, Xu *et al.*

Organic electrosynthesis, which employs electrons as reagents,^81^ has been demonstrated to be a versatile and environmentally friendly tool for the synthesis of structurally complex compounds.^82^ Lu, Xu and coworkers developed an electrochemical method for generating amidyl radicals and demonstrated that these species could participate in cascade cyclization reactions^83^ to afford indolines.^84^

**Scheme 13:**
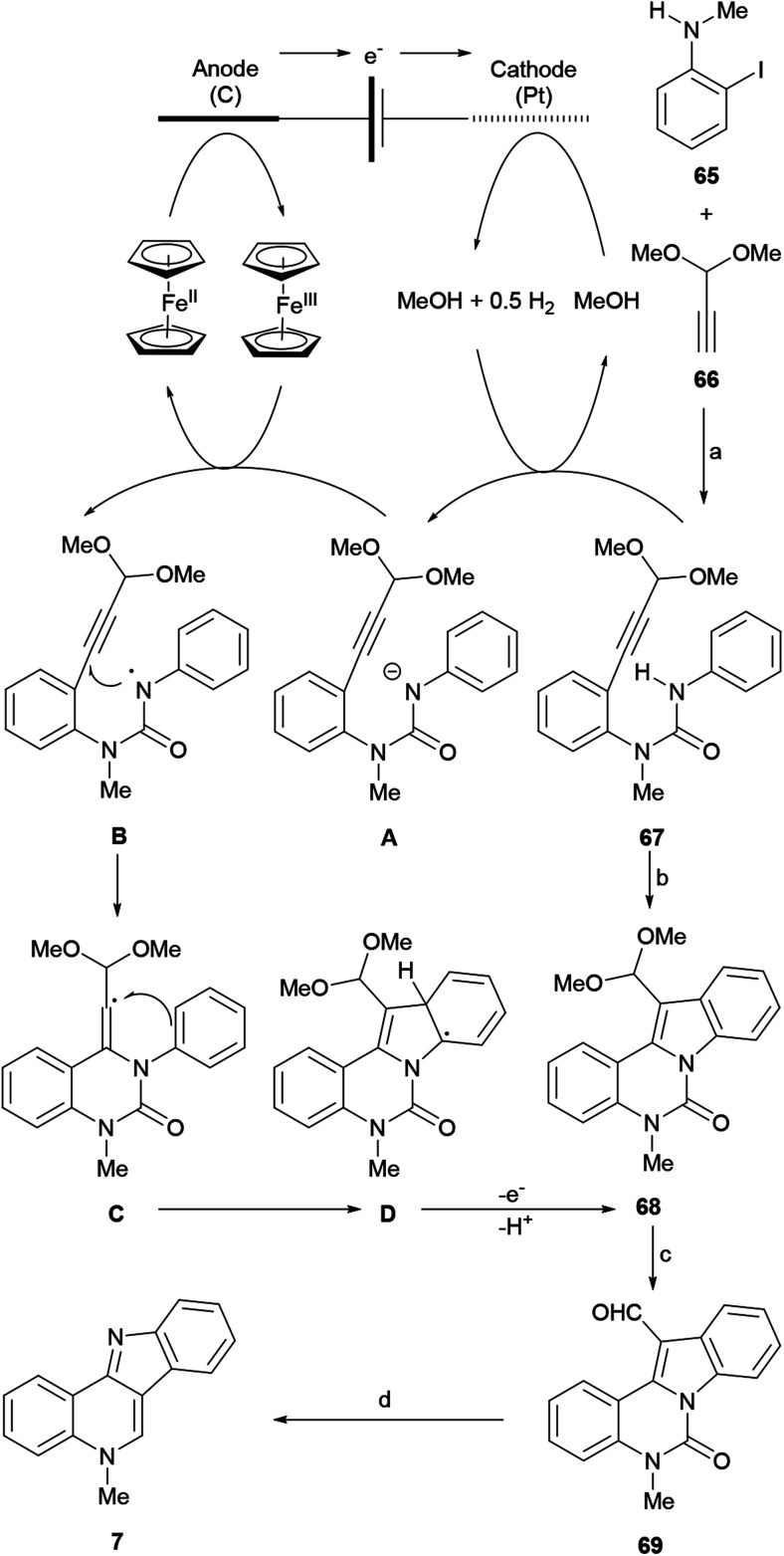
Reagents and conditions: (a) (1) Pd(Ph_3_P)_2_Cl_2_, CuI, Et_3_N : THF (1 : 1, v/v), rt, 12 h (81%); (2) PhNCO, Et_3_N, PhMe, rt, 48 h (78%); (b) Cp_2_Fe, MeOH : THF (1 : 5, v/v), Na_2_CO_3_, ^*n*^Bu_4_NBF_4_, reflux, 20 h; electrolysis at constant current (5 mA) with a reticulated vitreous carbon anode, Pt cathode; (c) 0.2 N HCl, rt, 30 min (63% overall); (d) NaOH, EtOH, reflux, 2 h (91%).

Their approach was applied in 2016 to the synthesis of highly functionalized indoles, including isocryptolepine, *via* C–H/N–H functionalization of (hetero)-arylamines with tethered alkynes.^85^ The synthesis (Scheme 13) commenced with a Sonogashira cross-coupling between *N*-methyl-2-iodoaniline (65) and the acetylenic acetal 66 in Et_3_N–THF at room temperature. The subsequent reaction of the resulting intermediate, obtained in 81% yield, with phenylisocyanate in toluene, gave the urea 67 in 78% yield.

Compound 67 became a suitable substrate for the electrolysis, which was carried out at a constant current of 10 mA on the gram-scale, in the presence of ferrocene as a redox relay reagent. The electrolyte was a mixture of sodium carbonate and tetrabutylammonium tetrafluorborate in THF–MeOH. A platinum plate was used as a cathode and reticulated vitreous carbon served as an anode.

This process afforded the tetracyclic acetalic compound 68, which after its subsequent acid-promoted hydrolysis resulted in the formation of the formyl-substituted indole 69 in 63% overall yield. Compound 69 was readily transformed into isocryptolepine (7) in one step and 91% yield, *via* the base-promoted hydrolysis of the urea motif, which freed the *N*-methylaniline moiety, enabling its spontaneous intramolecular condensation with the free formyl group. Among the advantages of this approach are the facts that it proceeds through the liberation of H_2_ and does not require the use of noble-metal catalysts and external oxidants for the cyclization stages.

A plausible mechanism for the electrochemical formation of the product was proposed based on previous studies.^84^ Accordingly, the anodic oxidation of [Cp_2_Fe] to [Cp_2_Fe]^+^ takes place with the concomitant cathodic reduction of MeOH to form the methoxide anion and release H_2_. Then, the base deprotonates the amino group of the substrate 67 to furnish the anion A, which is a much better electron donor than its neutral precursor.

Therefore, single-electron transfer (SET) takes place between intermediate A and [Cp^2^Fe]^+^ to afford the electron-deficient nitrogen-centered radical B^86^ and regenerate Cp_2_Fe.^87^ It was suggested that the SET is so efficient, that it competes well with the cathodic reduction of [Cp_2_Fe]^+^, enabling the electrolysis to be executed in an undivided cell. Next, the intermediate B participates in a rare 6-*exo-dig* cyclization^83^ to give the vinyl radical C, which then undergoes a second cyclization with the aryl ring, to afford the delocalized tetracyclic radical D. Finally, the rearomatization of the latter by electron and proton eliminations generates the product 68.

#### Synthesis by Aksenov *et al.*

The 2009 synthesis of isocryptolepine by Kundu and co-workers comprised four steps, which involved a Fischer indolization to build a suitable 2-arylindole intermediate and the installation of the quinoline ring *via* an oxidative Pictet–Spengler type reaction of an *N*-arylmethanimine, in ∼50% overall yield. Shortly after, Aksenov and coworkers developed a sequential three-component heteroannulation reaction toward 3-aryl-2-quinolones that proceeds *via* a cascade transformation and involves a Fisher indole synthesis with PPA as the reaction medium. The authors observed that the P_2_O_5_–water ratio can be accurately tuned in the promoter to provide optimal yields of the cyclized product, and proposed to use their development for a simplified and improved total synthesis of isocryptolepine, under the original retrosynthetic guidelines of Kundu.^88^

**Scheme 14:**
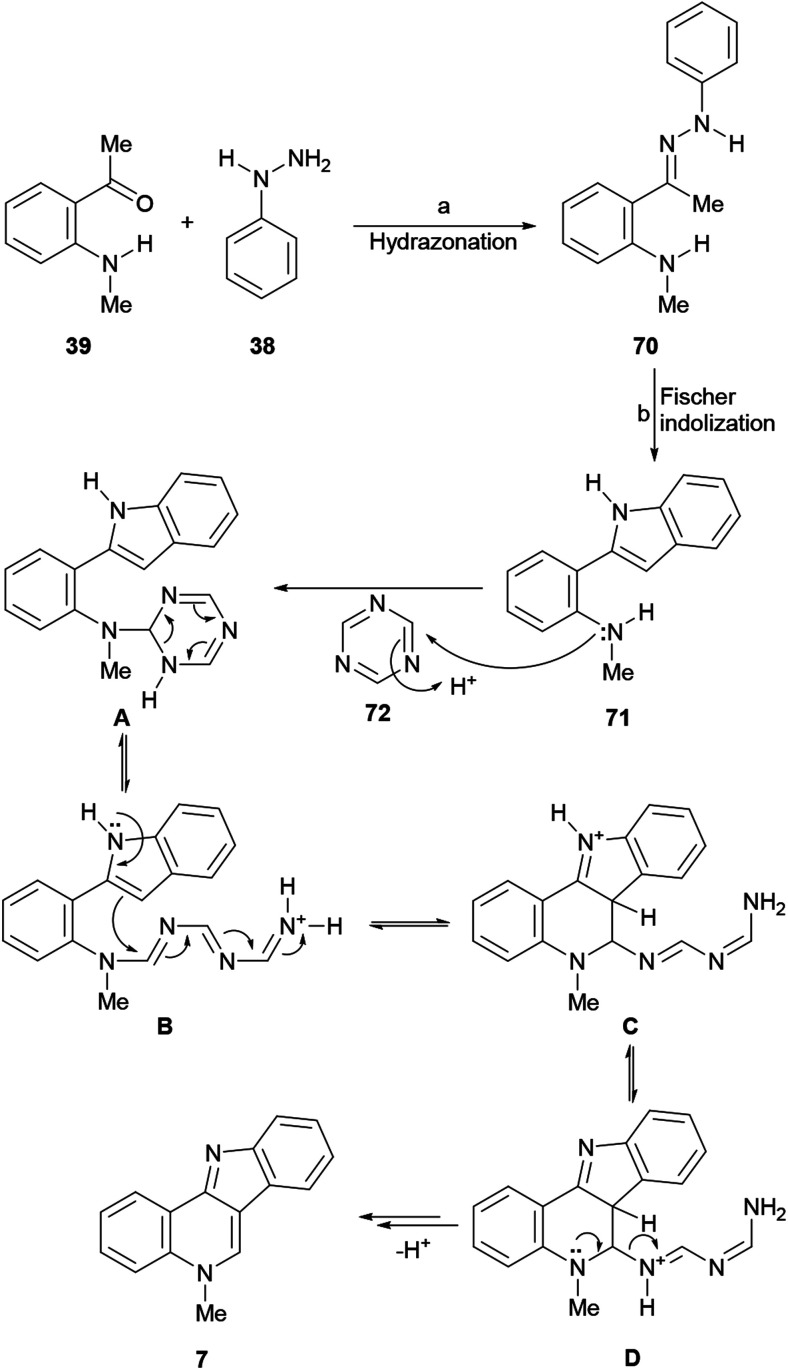
Reagents and conditions: (a) HCO_2_H (cat.), EtOH, reflux, 30 min, or neat, 120 °C, 20 min; (b) 80% PPA, 80 °C, 2 h; 130 °C, 90 min (83%).

In that direction, in 2017 Aksenov *et al.* conjectured that PPA could promote a one pot synthesis of the intermediate hydrazone 70 required^89^ for Fischer indolization (Scheme 14), as well as drive to completion the formation of the indole moiety (71), and also participate in the subsequent intramolecular Vilsmeier reaction between an aromatic amine and a carboxylic acid, for the final cyclization leading to isocryptolepine (7).^90^

However, PPA failed to promote the synthesis of the hydrazone between *ortho*-aminoacetophenone (39) and phenylhydrazine (38), favoring self-condensation of the ketone, and this step had to be performed in EtOH under formic acid catalysis^91^ or even better, under solventless conditions, for 10 min at 120 °C. Furthermore, despite PPA was able to promote the Vilsmeier cyclization with various carboxylic acids, but formic acid proved to be unstable to the reaction conditions, decomposing into CO and CO_2_ and furnishing 7 in only 25% yield.^92^

Therefore, a formic acid surrogate was sought and triazine was proposed to fulfill this place. The authors found that once hydrazone 70 was formed, it could be treated with PPA to afford indole 71, and shortly after with triazine (72) to produce isocryptolepine as the sole reaction product in an optimized 82% yield, using a convenient metal-free process.

The proposed reaction mechanism of the transformation involving triazine as a formic acid equivalent comprises the addition of the triazine (72) to the Fischer indolization product 71 to produce species A. Next, ring opening of the dihydrotriazine and subsequent intramolecular electrophilic attack of the resulting linear species B at C3 of the indole furnishes the six-membered heterocyclic ring (C). Finally, proton transfer, followed by an elimination (D) anchimerically-assisted by the quinoline nitrogen, affords isocryptolepine. An alternate deprotonation/elimination sequence leading to 7 has also been proposed. In addition, the same group devised a second generation PPA-mediated one-pot three-component synthesis of the indoloquinoline scaffold employing nitroalkanes. The use of MeNO_2_ in place of the triazine 72 furnished 7.^93^

Interestingly, the use of dimethyl sulfoxide (DMSO) as a one-carbon atom synthon in a similar transformation leading to the unsubstituted indoloquinoline skeleton in 69% yield was recently reported.^94^ In this case, it was proposed that two mechanistic pathways are possible. In the first case, at high temperature, DMSO may generate methyl radicals (Me˙), which can react with O_2_ to produce a peroxy radical and then CH_2_O.^95^ In turn, this could result in imine formation and a subsequent electrophilic aromatic substitution to produce the observed product. It is also possible that DMSO could decompose at high temperature, and produce CH_2_O directly.^96^

### 4c. Syntheses from quinolines

The total syntheses of isocryptolepine, which employs quinolines as starting materials, comprise synthetic efforts where haloquinolines or 1,2,3,4-tetrahydroquinolin-4-one derivatives were employed. They complement other accomplishments, such as that of Bogányi and Kámán,^76^ where quinolines were prepared as intermediates from structurally simple benzenoids.

#### Synthesis by Tímari *et al.*

**Scheme 15:**
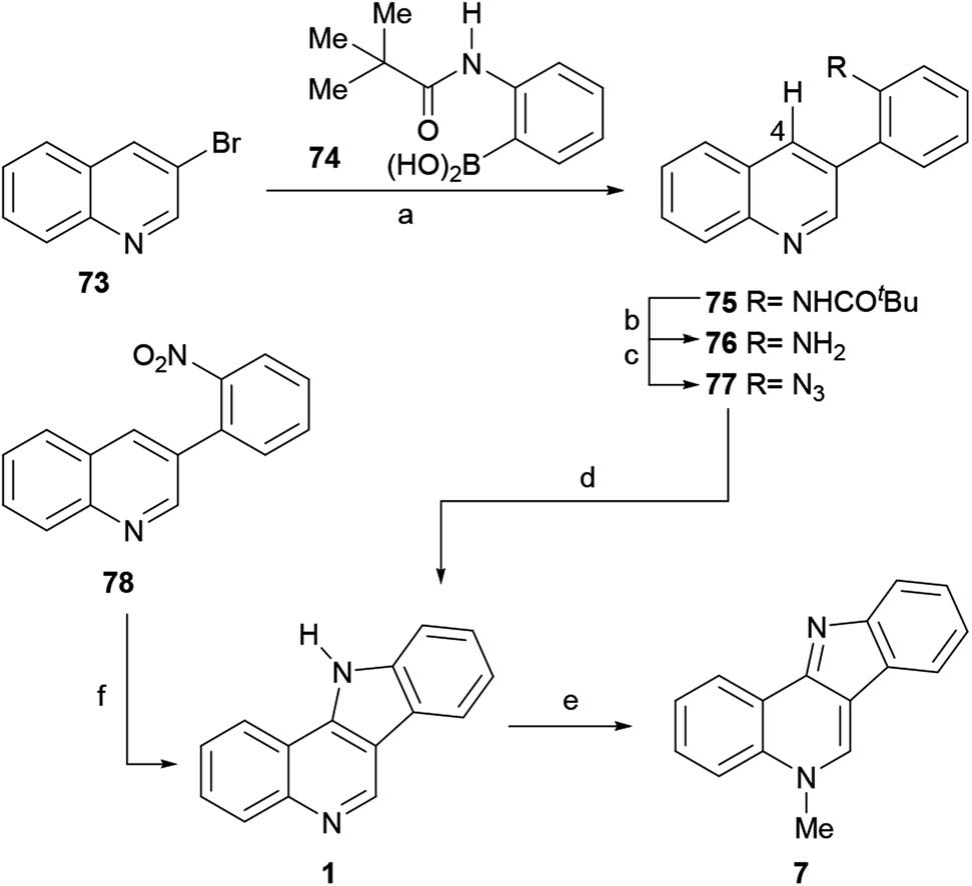
Reagents and conditions: (a) Pd(0), DME, H_2_O, NaHCO_3_, reflux, 4 h (90%); (b) 20% H_2_SO_4_, reflux, 24 h (93%); (c) (1) NaNO_2_, HCl (conc.), 0 °C, 1 h; (2) NaN_3_, 0 °C, 1 h (80%); (d) 1,2-Cl_2_C_6_H_4_, 180 °C, 5 h (75%); (e) (1) Me_2_SO_4_, CH_3_CN, reflux, 5 h; (2) K_2_CO_3_, H_2_O (93%); (f) PhMgBr, THF, 0 °C, 15 min (46%).

The first total synthesis of isocryptolepine starting from quinolines was reported in 1997 by the Tímari group (Scheme 15).^97^ The sequence resorted to a Suzuki cross-coupling^98^ reaction between *N*-pivaloylamino phenylboronic acid (74)^99^ and 3-bromoquinoline (73) to afford the biaryl derivative 75 in 90% yield. Next, the acid-catalyzed removal of the protecting group furnished 93% yield of the free amine 76, which was subjected to a one-pot diazotization and subsequent reaction with NaN_3_ to deliver the azide derivative 77 in 80% overall yield.^100^

The thermal cyclization of 77 was executed in refluxing dichlorobenzene, and proceeded in 70% yield to exclusively furnish the required tetracycle 1 in 75% yield,^101^ by way of a singlet nitrene intermediate that inserts into the C4–H bond. The reaction of 1 with Me_2_SO_4_ in MeCN resulted in the regioselective alkylation of the pyridine nitrogen, affording isocryptolepine (7) in 93% yield after freeing the base by treatment with potassium carbonate.

An alternate pathway for accessing 1 in one pot and 46% yield from 78 was recently disclosed by Ess, Kürti and coworkers, through the electrophilic amination of the quinoline C4–H bond with PhMgBr.^102^ On the other hand, the Sydnes group disclosed the preparation of tetracycle 1 from the aniline derivative 76 in 73% yield, employing a palladium-catalyzed C–H activation/C–N bond formation strategy.^103^

#### Syntheses by Maes *et al.*

**Scheme 16:**
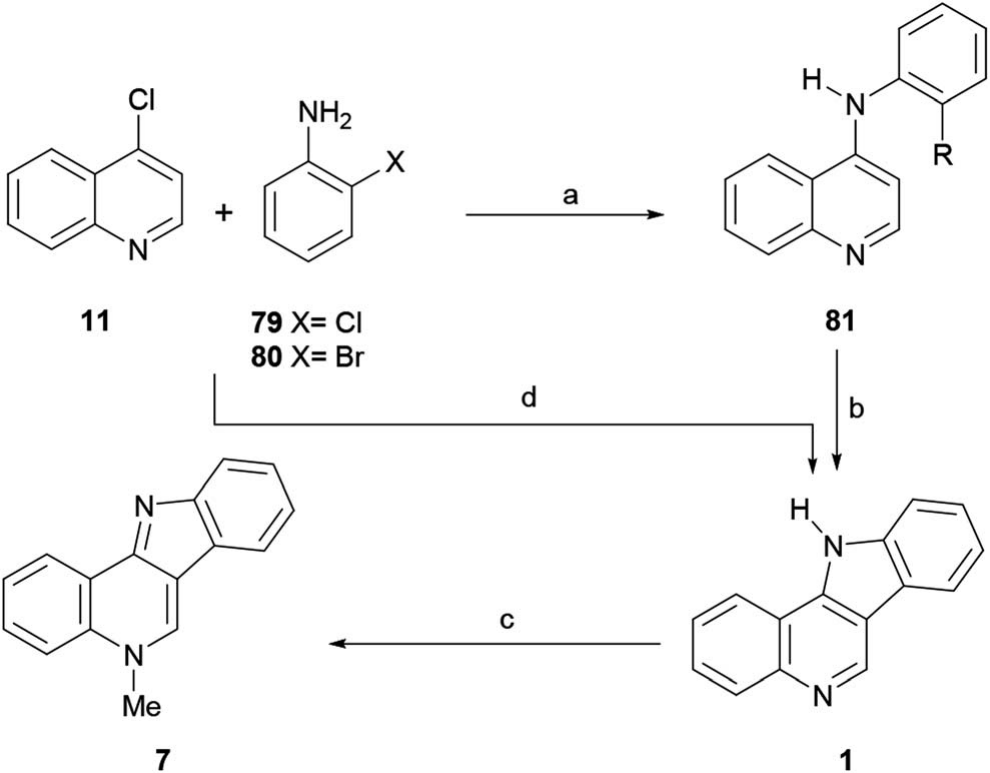
Reagents and conditions: (a) Pd(OAc)_2_, BINAP, K_2_CO_3_, dioxane, reflux, overnight (60%), or Pd_2_(dba)_3_, Xantphos, Cs_2_CO_3_, dioxane, reflux, overnight (81%); (b) Pd_2_(dba)_3_, P(*t*-Bu)_3_, K_3_PO_4_, dioxane, 120 °C (closed vessel), 3 h (95%); (c) (1) CH_3_I, DMF, 80 °C, 1 h; (2) overnight, r.t.; (3) aq. Na_2_CO_3_ (57%); (d) Pd_2_Cl_2_(dba)_3_ (5 mol%), P(Bu)_3_ (20 mol%), K_3_PO_4_ (20 mol%), dioxane, 125 °C closed vessel, 24 h (82%, from 79, R = Cl) or PdCl_2_(PPh_3_)_2_ (1 mol%), NaOAc·3H_2_O (1.47 mol%), DMA, MW, 200 °C, 10 min (79%, from 80, R = Br).

In 2003, the Maes group^104^ developed a three-step synthesis of isocryptolepine (Scheme 16) based on the combination of two selective Pd-catalyzed transformations, namely a Buchwald–Hartwig reaction^105^ and an intramolecular direct arylation.^106^ Thus, the arylamination of 4-chloroquinoline^107^ (11) and 79 with Pd(OAc)_2_ and K_2_CO_3_ as a base, in refluxing dioxane, afforded the amine 81 in 60% yield when BINAP was employed as the metal ligand.

However, the use of Xantphos as a ligand improved the yield to 81% when Cs_2_CO_3_ was employed as a base.^108^ In order to execute the required direct arylation of 81, a nucleophilic palladium catalyst system better than Pd/BINAP was deemed necessary.^109^ Therefore, trialkylphosphine ligands, which are more electron rich than their triaryl counterparts, were examined, with the finding that the cyclization could be carried out to afford a 95% yield of 1 when tributylphosphine was used,^110^ in the presence of potassium phosphate.

The direct arylation leading to 1 was considered a notable example of the intramolecular Pd-catalyzed arylation of an electron-deficient heteroaromatic. Examples of these reactions are scarcer in the literature compared to those of electron-rich heteroarenes.^111^ Final, methylation of 1 with MeI in DMF, followed by treatment with Na_2_CO_3_ to release the base, afforded isocryptolepine (7) in 57% yield.

The synthetic strategy was further improved by these authors in 2008,^112^ with the development of an auto-tandem palladium-catalyzed consecutive C–N and C–C bond forming process, which enabled the coupling of 4-chloroquinoline (11) and 2-chloroaniline (79), somehow reminiscent of the auto-tandem syntheses of other carbazole systems.^106*c*,113^ Employing the catalytic system previously used for the direct arylation [Pd_2_Cl_2_(dba)_3_, P(^*t*^Bu)_3_], with K_3_PO_4_ as a mild base for improved compatibility, the tetracyclic compound 1 was obtained in 82% yield. It was also observed that despite K_3_PO_4_ not dissolving completely in the reaction medium, the addition of an excess of reagent can speed-up the reaction.

#### Syntheses by Mohan *et al.*

**Scheme 17:**
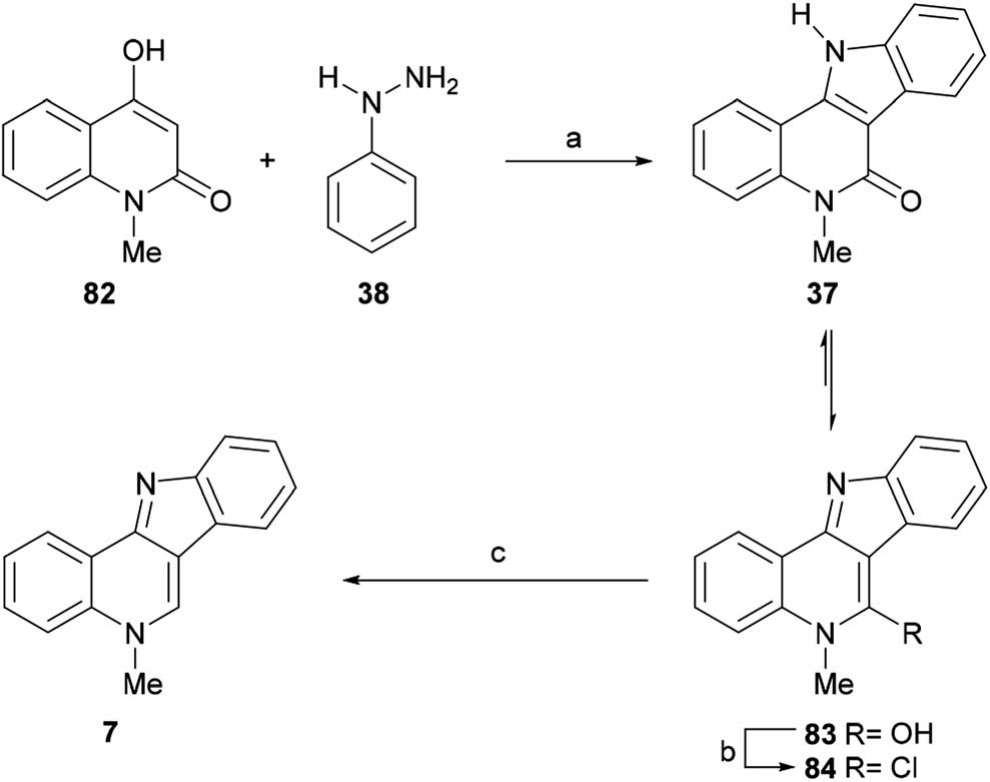
Reagents and conditions: (a) AcOH_gl_/12 N HCl (4 : 1), 135 °C, 5 h (65%); (b) POCl_3_, 100 °C, 8 h (62%); (c) H_2_, 10% Pd/C (70%).

The Mohan group achieved many advances in the synthesis of indoloquinolines, with two of their total syntheses of isocryptolepine employing quinoline derivatives as starting materials. In 2005 they reported the first of these total syntheses, which was based on the Fischer indole cyclization of the 1,3-dicarbonyl derivative 82 (Scheme 17).^114^

To that end, 82 was exposed to phenylhydrazine hydrochloride (38), in a 4 : 1 mixture of glacial AcOH and concentrated HCl, to give the indoloquinolinone 37 in 65% yield. The latter existed predominantly in the hydroxy form (83); therefore, the required aromatizative deoxygenation of 83 was performed as a two-step process, by reaction with POCl_3_ (ref. 115) to easily form the intermediate chloro-derivative 84 (62% yield), which was hydrogenolyzed over 10% Pd/C to give isocryptolepine (7) in 70% yield.

The authors observed that the yield of product for the photocyclization in protic solvents was significantly higher than that in benzene alone; hence, the reactions were carried out in a benzene : methanol : sulfuric acid (60 : 30 : 1, v/v/v) solution, to afford the tetracycle 1 in 78% yield.^10*b*^ The final *N*-methylation of 1 was performed with methyl sulfate in acetonitrile, furnishing isocryptolepine (7) in 83% yield after freeing the base with K_2_CO_3_. Furthermore, a similar approach culminating with the photoannulation of a 3-iodoquinoline derivative was employed by this group for the synthesis of a 6-methyl derivative of isocryptolepine.^116^

**Scheme 18:**
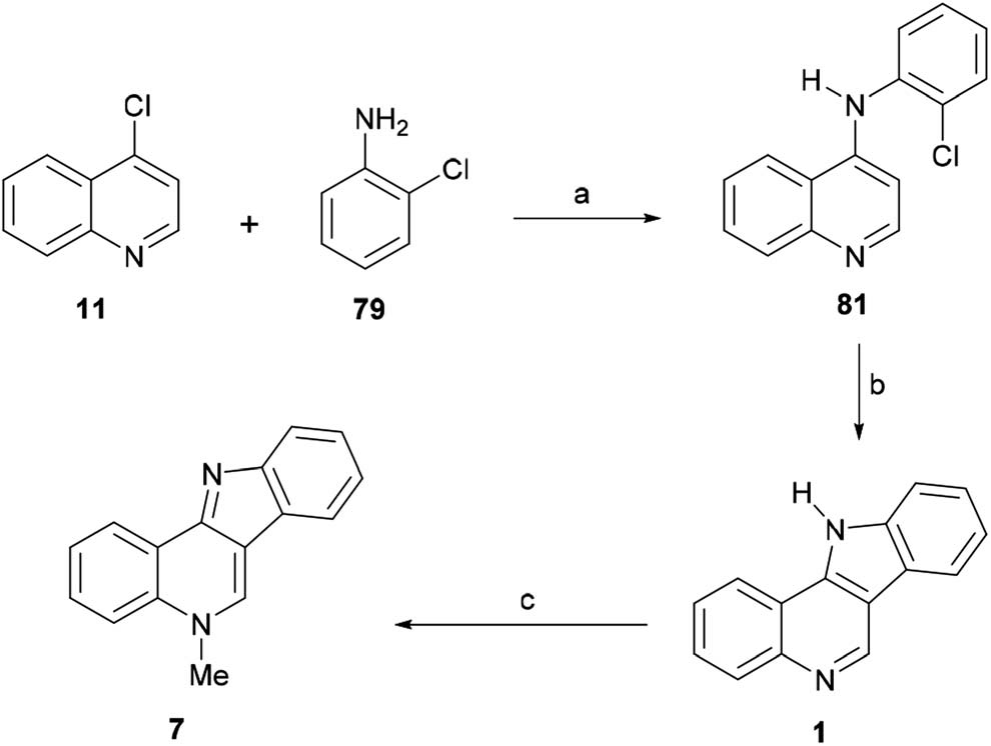
Reagents and conditions: (a) 200 °C, 5 h (74%); (b) *hν*, PhH : MeOH : H_2_SO_4_ (60 : 30 : 1, v/v/v), I_2_, rt (78%); (c) Me_2_SO_4_, MeCN, reflux, 6 h, K_2_CO_3_ (83%).

In 2006, the same group reported an efficient three-step heteroatom directed photoannulation approach toward the natural product (Scheme 18).^10*b*^ In their synthetic sequence, 4-chloroquinoline (1) was thermally aminated at 200 °C in 74% yield with 2-chloroaniline (79),^117^ and the resulting 4-anilinoquinoline derivative 81 (also prepared by Maes *et al.*) was submitted to an oxidative photoannulation in various solvents and in the presence of iodine, as a mild oxidant.

#### Synthesis by Kumar *et al.*

**Scheme 19:**
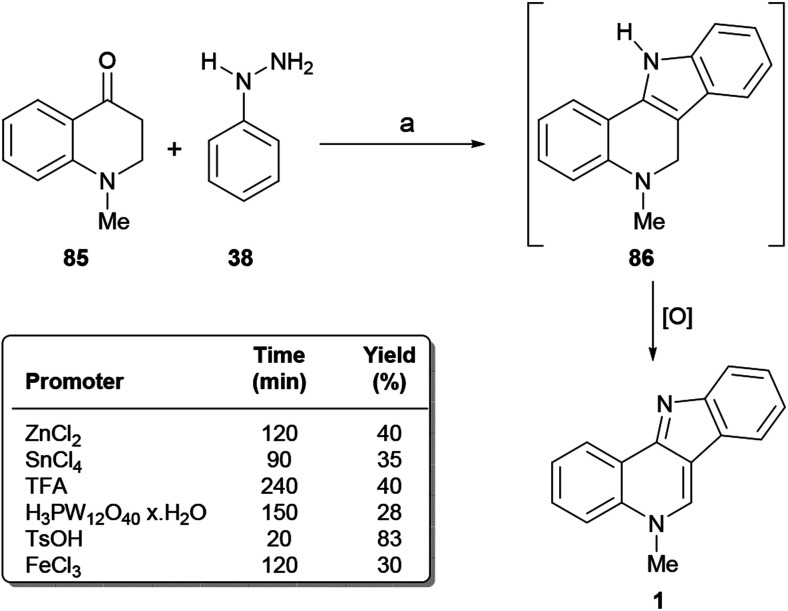
Reagents and conditions: (a) Promoter (1.1 equiv.), neat, air, 100 °C. Reaction times and overall yields are shown in the table.

Inspired by a strategically similar approach to 1,2,3,4-tetrahydroisoquinolines,^118^ in 2009 the Kumar group disclosed (Scheme 19) an interesting solvent-free^119^ variation of one of the syntheses of Mohan,^114^ and the early work of Braunholtz and Mann,^45*a*^ leading to isocryptolepine. The sequence features the one step Fischer indolization-aerobic oxidation of *N*-methyl dihydroquinolin-4-one 85 with phenylhydrazine (38) under acid promotion.^120^

The transformation involves the cyclocondensation toward the tetracycle 86 and its further dehydrogenation *in situ*^10*b*,89*a*,121^ to afford the final product. The authors tested different alternative acidic promoters, discovering that heating with a 1.1 equiv. TsOH at 100 °C gave the best result (83% yield).

### 4d. Syntheses from indoles

The approaches to the total synthesis of isocryptolepine from indoles are characterized by the use of *N*-protected and/or 3-substituted indoles as starting materials. Although certain syntheses have used an unprotected indole derivative and others have started with 2-substituted indoles, they are fewer in number. Electron withdrawing groups (benzenesulfonyl, Boc) do not always meet C–C or C–N bond formation requirements; in such cases protective groups such as SEM, MOM and benzyl ether have been used.

#### Synthesis by Joule *et al.*

**Scheme 20:**
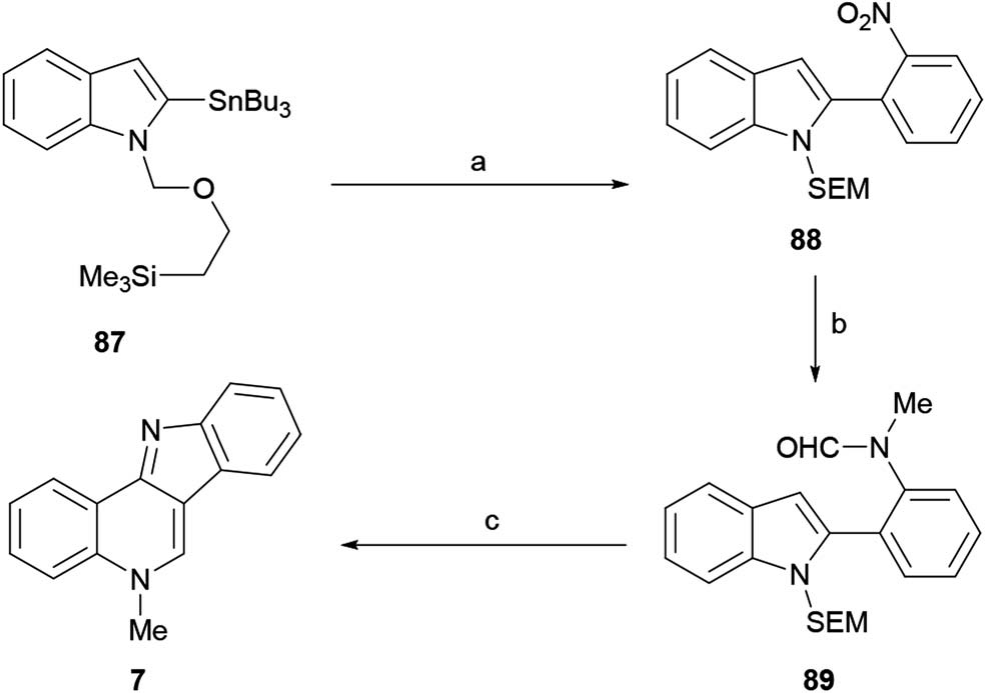
Reagents and conditions: (a) Pd(PPh_3_)_4_, 2-IC_6_H_4_NO_2_, THF, reflux (98%); (b) (1) H_2_, Pd/C, EtOH, rt (98%); (2) AFA, THF, −20 °C; (3) NaH, THF, rt; (4) MeI (95% overall); (c) EtOH, 10% H_2_SO_4_, reflux (50%).

The first indole-based total synthesis of isocryptolepine was reported in 1998 by the Joule group, as a different approach toward the natural product (Scheme 20). The sequence employed the indole-stannane derivative 87 as a starting material and resorted to an intramolecular Vilsmeier reaction to build the 3-aminoalkylidene-3*H*-indole motif of the natural product.^122^

Therefore, the 2-tributylstannyl indole 87 was *N*-protected as the SEM derivative and coupled with 2-iodonitrobenzene under palladium catalysis, to give the 2-(*ortho*-nitrophenyl)indole 88 in 98% yield. The catalytic hydrogenation of the nitro group (98% yield), followed by *N*-formylation with acetic formic anhydride (AFA)^123^ and subsequent *N*-methylation of the resulting amine with methyl iodide, gave 89 in 95% overall yield.

The latter was subjected to an intramolecular Vilsmeier ring closure with sulfuric acid in ethanol, to afford 7 in 50% yield. Interestingly, protection of the starting indole as the *N*-phenylsulfonyl derivative produced complex mixtures, presumably due to the deactivating effect of the sulfonyl moiety toward the final electrophilic attack.

#### Synthesis by Mohan *et al.*

**Scheme 21:**
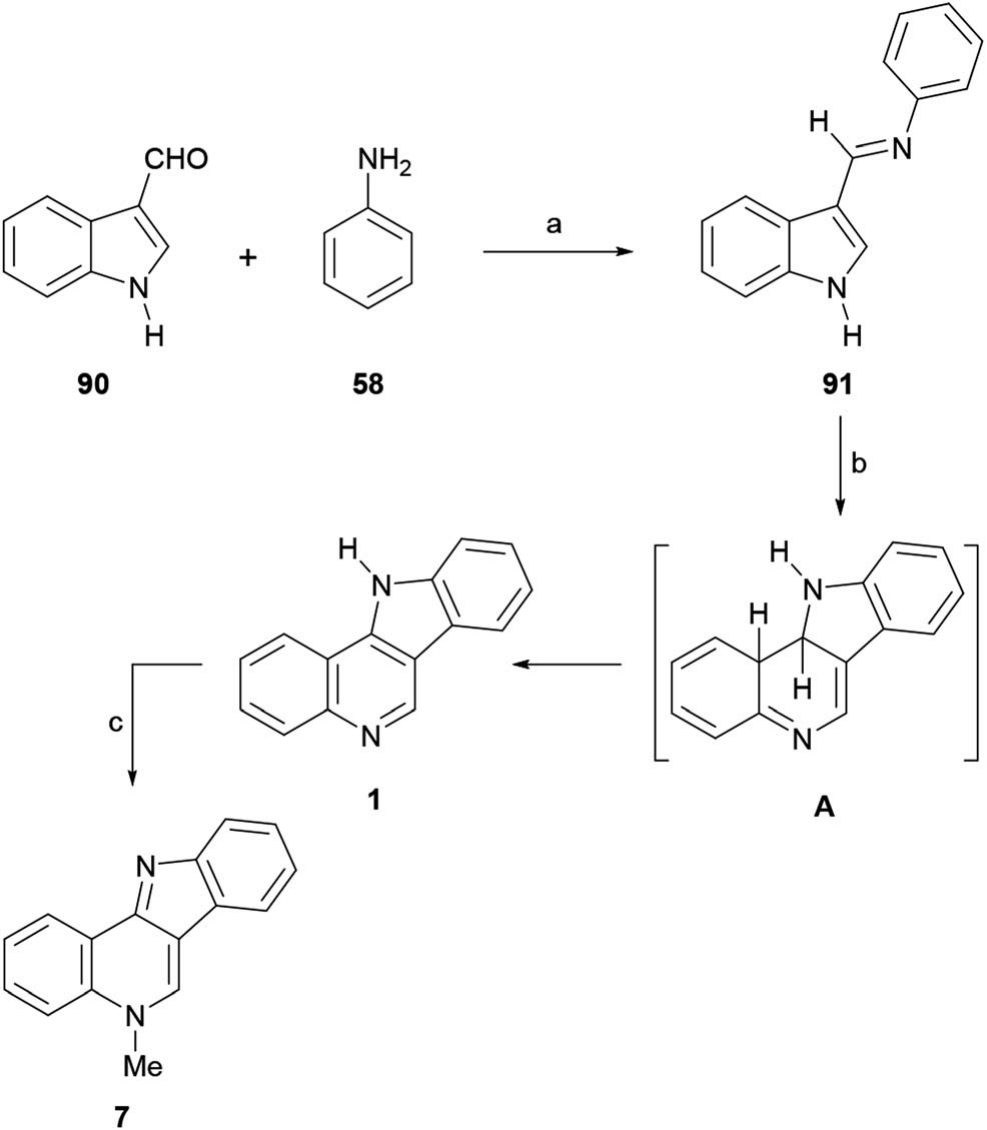
Reactions and conditions: (a) AcOH_gl_, reflux, 3 h (85%); (b) (1) *hν* (254 nm), PhH–MeOH, rt, 2 : 1; (2) I_2_, 48 h (67% overall); (c) Me_2_SO_4_, MeCN, reflux, 6 h, K_2_CO_3_ (83%).

The Mohan group developed photochemical approaches to the synthesis of several heteroannelated acridines,^124^ including indoloacridines.^125^ In 2002, they extended their strategy to the total synthesis of isocryptolepine.^89*a*^ The synthetic sequence employed simple and readily available starting materials, and involved the reaction of indole-3-carbaldehyde (90) with aniline (58) in glacial AcOH to afford 85% yield of the Schiff base 91, which exhibits a 1,3,5-hexatriene motif (Scheme 21). Then, 91 was dissolved in benzene-methanol and exposed to short wavelength UV-light (254 nm) in the presence of iodine, to give the tetracyclic system 1.^126*a*^ It is worth noting that a visible-light-mediated aerobic tandem dehydrogenative Povarov/aromatization reaction with iodine^126*b*^ for the synthesis of isocryptolepines has been recently reported and the use of indole-3-carbaldehyde (90) as a precursor for the synthesis of bioactive alkaloids has been recently reviewed.^127^

Mechanistically, 91 first underwent photo-isomerization of the *E*-isomer to its *Z*-congener; then, the excited S1 state of the *Z*-isomer underwent an orbital symmetry-controlled conrotatory ring closure to the tetracyclic intermediate A.

These types of dihydro intermediates are sensitive to oxygen;^121^ therefore, A underwent aromatization by way of the loss of the allylic hydrogens to form 1 in 67% yield, in the presence of iodine which acted as a mild oxidizing agent.

The final regioselective methylation of 1 with Me_2_SO_4_ in MeCN, in the presence of K_2_CO_3_, gave isocryptolepine in 83% yield. This total synthesis was complemented in 2006 with a three step photoannulation approach, which started from a quinoline derivative.^10*b*^

#### Synthesis by Miki *et al.*

**Scheme 22:**
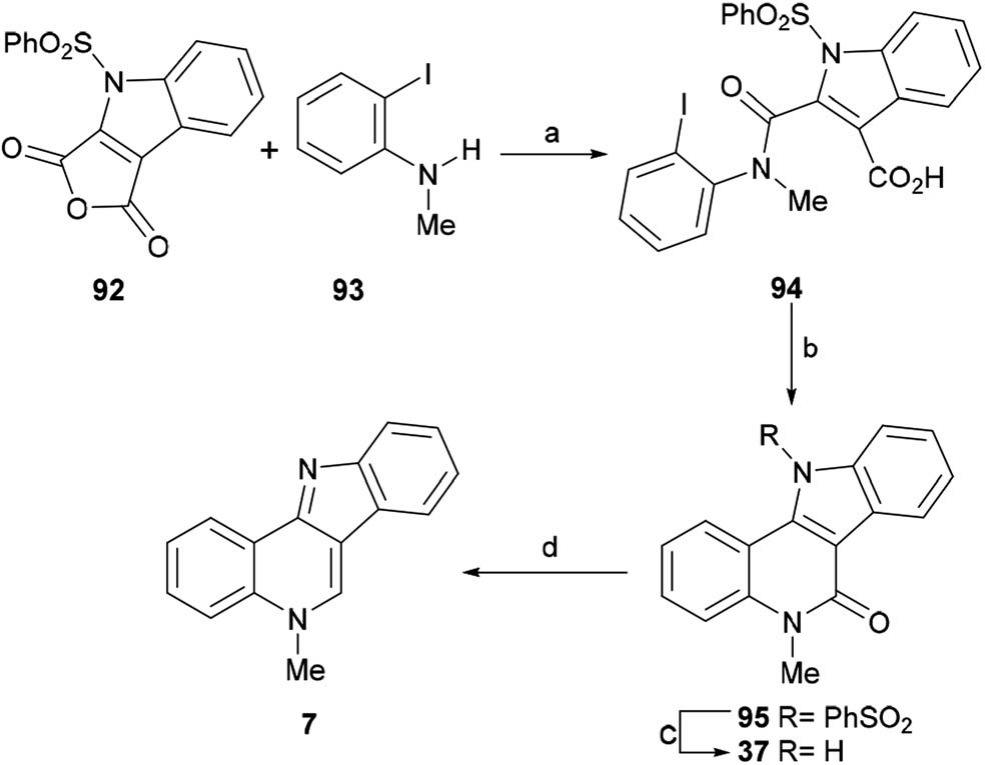
Reagents and conditions: (a) MeCN, rt, 72 h (77%); (b) Pd(TFA)_2_, Ag_2_CO_3_, 5% DMSO, DMF, 50 °C, 48 h (50%); (c) TBAF, THF, rt, 6 h (80%); (d) LiAlH_4_, dioxane, 1 h (98%).

The Miki group observed that indole-2,3-dicarboxylic anhydrides are useful synthons to access complex natural products.^128^ In 2007, they devised an intramolecular decarboxylative Heck-type reaction for the new total synthesis of isocryptolepine, employing this type of heterocycle (Scheme 22).^129^ In their approach, the anhydride moiety of the benzenesulfonyl derivative 92 was selectively opened upon exposure to *N*-methyl-2-iodoaniline (93) in MeCN at room temperature, to afford amide 94 in 77% yield.

Next, a projected decarboxylative Heck-type cyclization was executed following the protocol of Myers, by treatment of 94 with 20 mol% Pd(TFA)_2_ and Ag_2_CO_3_ in a mixture of 5% DMSO and DMF at 50 °C for 48 h,^130^ to give lactam 95 in 50% yield. Interestingly, a similar Heck cyclization was reported earlier by the Mérour group.^131^ In that process, lactam 95 was desulfonylated in 80% yield, by treatment with TBAF in THF at room temperature for 6 h,^54^ and the resulting tetracycle 37 was further reductively deoxygenated to isocryptolepine in 98% yield, by treatment with LiAlH_4_ in hot dioxane.

#### Synthesis by Hingane and Kusurkar

**Scheme 23:**
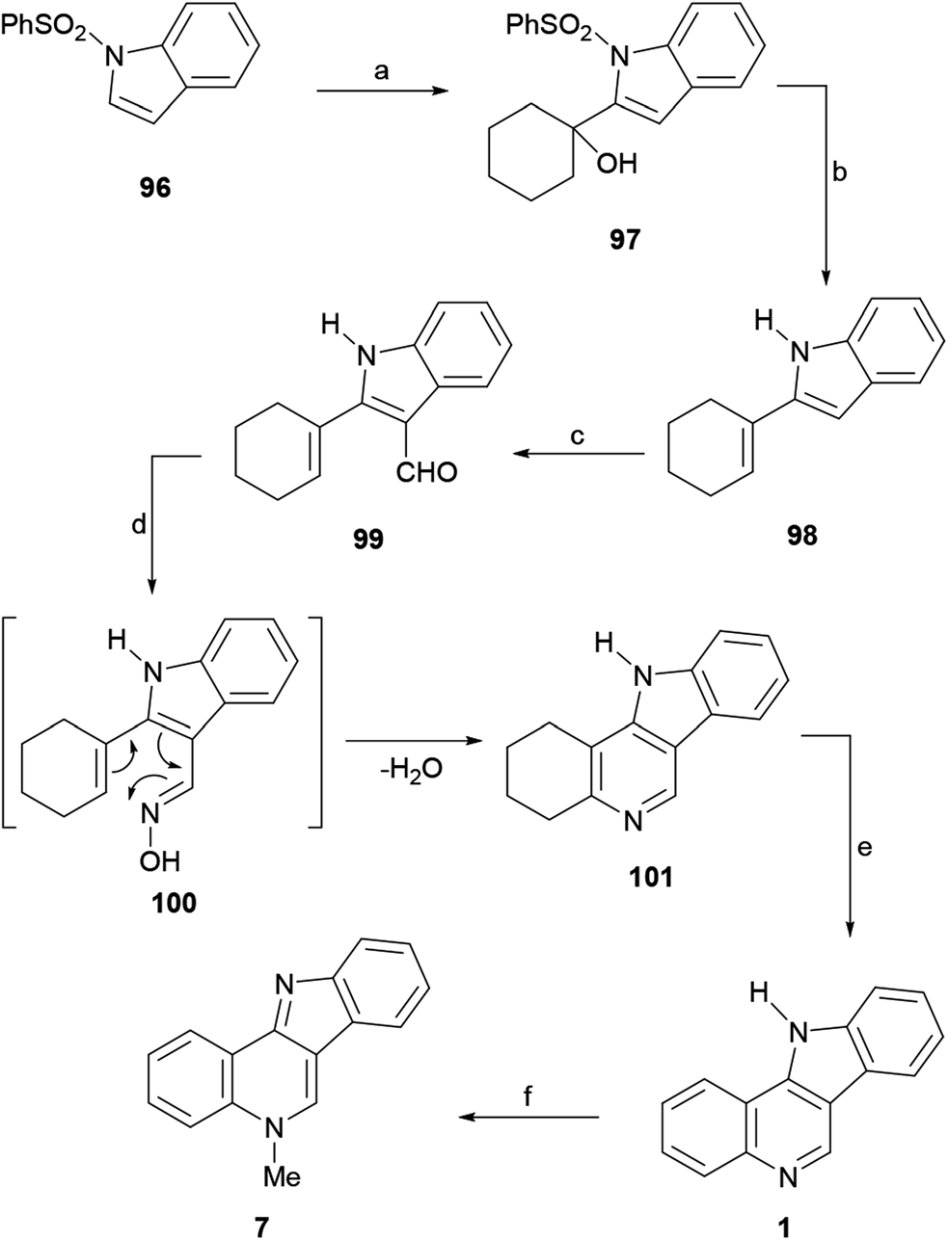
Reagents and conditions: (a) LDA, THF, −78 °C, cyclohexanone, 4 h (86%); (b) TBAF, THF, reflux, 2 h (84%); (c) POCl_3_, DMF, rt, 1 h (88%); (d) NH_2_OH·HCl, NaAcO, dioxane, reflux, 24 h (78%); (e) Pd/C, 1,2-Cl_2_C_6_H_4_, reflux, 20 h (88%); (f) (1) MeI, DMF, 80 °C, 1 h; (2) Na_2_CO_3_ (85%).

In 2011, Hingane and Kusurkar reported an imaginative 6π-electrocyclization approach toward isocryptolepine, starting from *N*-phenylsulfonyl indole (96).^132^ In their synthetic sequence, 96 was metallated with LDA and reacted with cyclohexanone to furnish the alcohol 97 (Scheme 23). Next, the hydrolytic *N*-desulfonylation of 97 was executed by treatment with TBAF in THF under reflux for 2 h, and took place with the concomitant dehydration of the tertiary alcohol moiety to afford 98 in 84% yield.^133^

The subsequent Vilsmeier–Haack formylation of 98 gave 99 in 88% yield, while attempted oximation of the latter with hydroxylamine in refluxing dioxane surprisingly furnished the tetracycle 101, presumably as a result of the initial formation of oxime 100 followed by a thermal intramolecular 6π-electrocyclization/elimination process. Therefore, the sequence continued with the dehydrogenation of 101 using Pd/C in *ortho*-dichlorobenzene, which afforded 1 in good yield. Finally, treatment of 1 with methyl iodide to afford the methiodide and sodium carbonate to free the base, gave 7 in 37% overall yield.

#### Synthesis by Hibino *et al.*

**Scheme 24:**
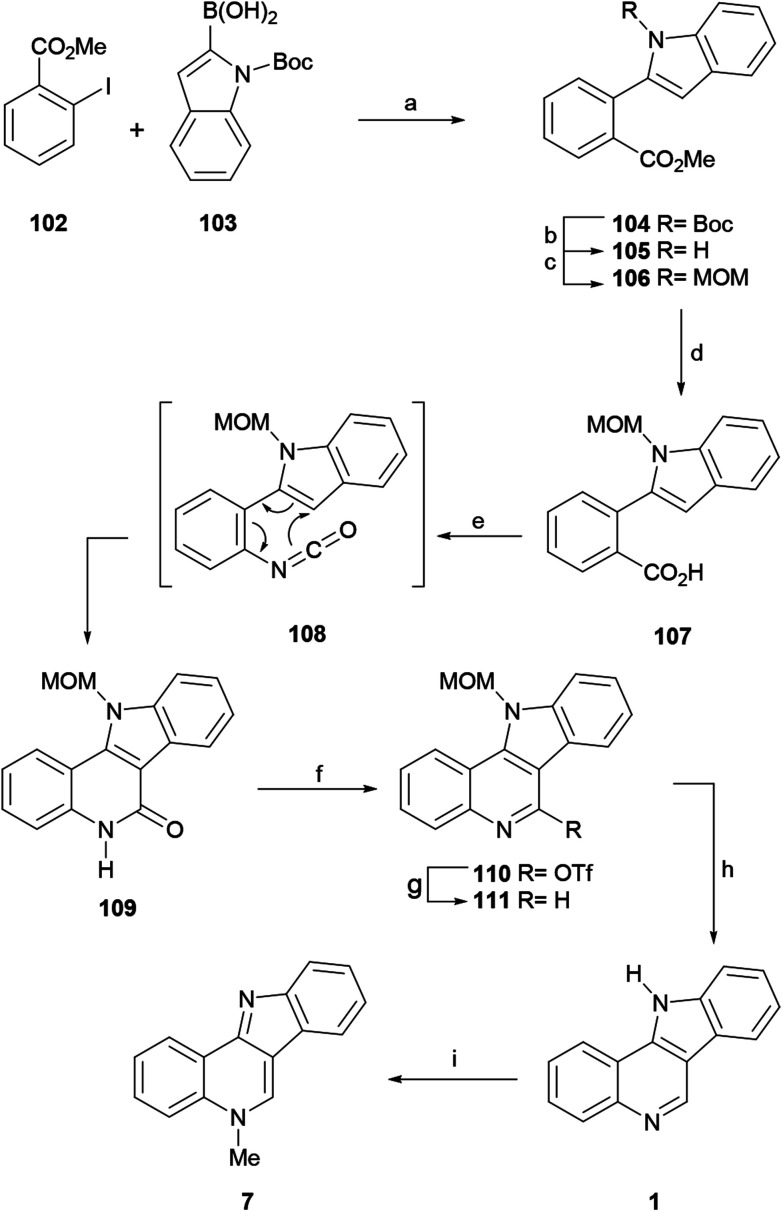
Reagents and conditions: (a) Pd(PPh_3_)_4_, Na_2_CO_3_, DME, reflux, 12 h (99%); (b) TFA, CH_2_Cl_2_, rt, 12 h (94%); (c) MOMCl, NaH, DMF, rt, 12 h (91%); (d) 1 M LiOH, THF, 60 °C, 12 h (98%); (e) DPPA, Et_3_N, PhMe, MW, 100 °C, 10 min (99%); (f) Tf_2_O, Py, CH_2_Cl_2_, rt, 30 min (83%); (g) Et_3_SiH, Pd(OAc)_2_, dppp, DMF, 60 °C, 1 h (91%); (h) MeOH, HC(OMe)_3_, CF_3_SO_3_H, MeNO_2_, 100 °C, 30 min (96%); (i) (1) MeI, PhMe, reflux, 2 h; (2) 28% NH_4_OH (90%).

In 2012, the Hibino group reported the total synthesis of isocryptolepine (7) *via* a microwave-assisted tandem Curtius rearrangement-electrocyclization of a 2-aza 6π-electron system (Scheme 24).^134^

Following the procedure of Beaumont, these authors subjected the 2-iodobenzoate 102 and the indole-2-boronic acid 103 to a Suzuki–Miyaura cross-coupling reaction and obtained 104 in 99% yield. Since Boc proved to be an unsuitable protecting group for the advanced stages of the synthesis, 104 was exposed to TFA, resulting in the deprotection of the *N*-Boc group in 94% yield (105).^135^ After trying different protecting groups, the authors selected the MOM group, which has minimum steric demand, to pursue the synthesis.

Hence, treatment of 105 with MOMCl in the presence of NaH gave the *N*-MOM-indolylbenzoate 106 in 91% yield and subsequent basic hydrolysis of the ester moiety with 1 M LiOH furnished the carboxylic acid 107 in 98% yield. The microwave-assisted Curtius rearrangement of the latter with DPPA in toluene at 100 °C quickly provided tetracycle 109, resulting from a subsequent one-pot thermal 6π-azaelectrocyclic reaction, in an optimized 99% yield, without the isolation of the isocyanate intermediate 108.

The deoxygenation of the lactam, required to complete the total synthesis, was achieved by treatment of 109 with triflic anhydride and pyridine, which afforded triflate 110 in 83% yield. Next, the subsequent reductive cleavage of the latter with Et_3_SiH under Pd catalysis, according to the procedure of Katsuki,^136^ provided the *N*-MOM-indoloquinoline 111 (91% yield), which was deprotected with triflic acid and trimethyl orthoformate in MeOH and nitromethane,^137^ to give indolo[3,2-*c*]quinoline 1 in 96% yield. The final methylation of 1 with MeI in toluene^59^ furnished isocryptolepine in 90% yield after release of the base.

#### Synthesis of Tummatorn *et al.*

Azides are known to easily extrude diatomic nitrogen and undergo migration of alkyl and aryl groups to the remaining electrophilic nitrogen atom. These migrations can be advantageously used to easily build structural complexity. The Pearson group demonstrated that the reaction of azides with electrophiles under acidic conditions gives iminium ions, which can be trapped by alkenes and alkynes.^138^

**Scheme 25:**
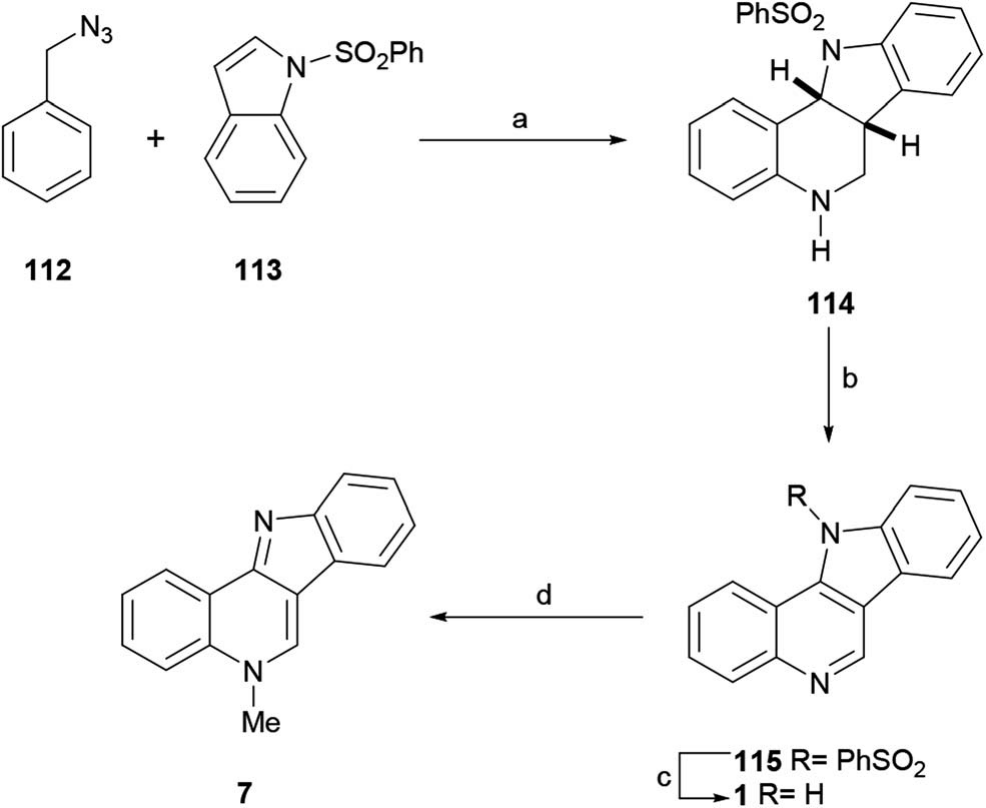
Reagents and conditions: (a) TfOH, CH_2_Cl_2_, rt, 14 h (94%); (b) DDQ, CH_2_Cl_2_, rt (94%); (c) 2 M NaOH, MeOH, reflux (95%); (d) (1) Me_2_SO_4_, MeCN; (2) K_2_CO_3_ (78%).

Speculating that the iminium ion could react with an aromatic nucleophile through an electrophilic aromatic substitution to afford a *N*-arylmethyl arene, in 2012 the Tummatorn group devised the four-step total synthesis of isocryptolepine (Scheme 25).^139^

In their synthetic sequence, benzyl azide (112) and *N*-phenylsulfonyl indole (113) were exposed to TfOH in CH_2_Cl_2_ to afford the tetracyclic compound 114 in 94% yield. Presumably, this reaction involved two sequential steps, namely the nucleophilic addition to the iminium ion followed by the intramolecular annulation of the resulting intermediate, resulting in a formal aza-[4 + 2]-cycloaddition.

Dehydrogenation of 114 with DDQ afforded 115 in 94% yield, which was desulfonylated in 95% yield with 2 M NaOH in refluxing MeOH to give the related indoloquinoline 1. The regioselective methylation of the latter with methyl sulfate finally furnished isocryptolepine in 78% yield. Functionalization of the precursor 114 has also been reported.^140^ On the other hand, the basic synthetic sequence was employed as a key strategy toward the development of analogs of the natural product.^141^

#### Synthesis by Lin *et al.*

Transition-metal catalyzed tandem C–C/C–N bond coupling reactions are an interesting strategy en route to nitrogenous compounds.^142^ In 2010, Jiao's group reported a Pd(ii)-catalyzed direct dehydrogenative annulation of indolecarboxamides with internal alkynes *via* C–H/N–H bond cleavage. Shortly after, in 2014, Cui and coworkers developed a Rh(iii)-catalyzed tandem coupling of *N*-methoxy-1*H*-indole-carboxamide and aryl boronic acids.^143^

The Lin group studied the palladium-catalyzed coupling of *N*-methoxycarboxamides with iodobenzene (117).^144^ They found that the reaction is capable of forming both C–C and C–N bonds in the presence of a suitable ligand, an oxidant and an indispensable silver source. PPh_3_ proved to be one of the best ligands to activate the metal,^145^ whereas silver carbonate proved to be the most appropriate source of silver, also acting as an oxidant.

**Scheme 26:**
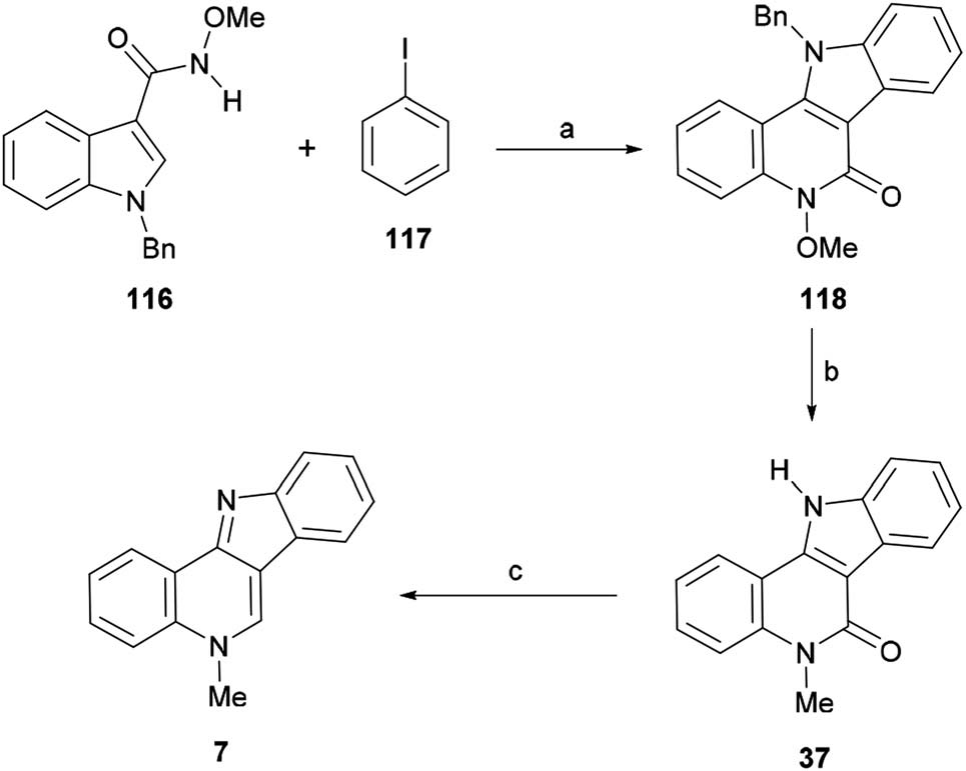
Reagents and conditions: (a) Pd(OAc)_2_ (10 mol%), PPh_3_ (20 mol%), Ag_2_CO_3_ (2 equiv.), AcOH, DMA (3 : 1, v/v), 100 °C, 6 h (67%); (b) (1) NaH, DMF, 120 °C, 30 min; (2) MeI, rt, 5 h; (3) NaH, 140 °C, 6 h (77%); (c) (1) NaBH_4_, BF_3_·Et_2_O THF, rt, 30 min; (2) reflux, 12 h (84%).

These authors applied the developed method to a new total synthesis of isocryptolepine (Scheme 26). Thus, the methoxyamide 116 was coupled with iodobenzene (117) under the optimized conditions to afford the tetracycle 118 in 67% yield. A mixed AcOH–DMA solvent was required in order to avoid decomposition of the starting material due to the considerable acidity of the solvent at the required high temperature.^146^

Next, the latter was exposed to MeI and an excess of NaH at elevated temperature, in order to achieve simultaneous debenzylation and selective *N*-methylation with concomitant demethoxylation and afford the key intermediate 37 in 77% yield.^147^ The final deoxygenation of the lactam in 37 was accomplished by partial reduction with NaBH_4_ in THF under the assistance of BF_3_·Et_2_O, followed by the *in situ* dehydration of the resulting lactol to afford 7 in 84% yield.

**Scheme 27:**
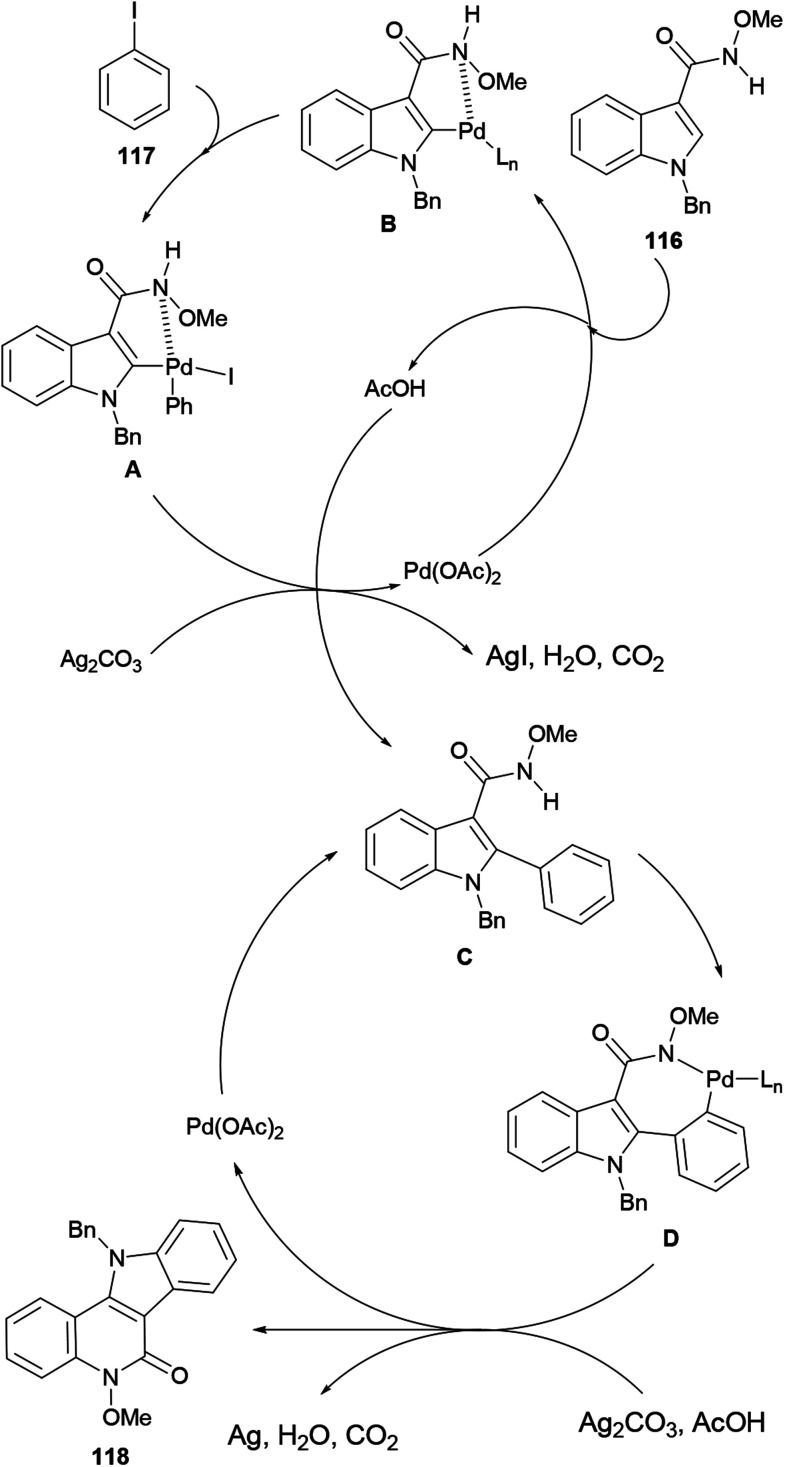
Proposed reaction mechanism for the tandem C–C/C–N bond formation reaction.

The reaction mechanism for the tandem C–C/C–N bond formation reaction (Scheme 27) involves the reaction of the starting material 116 with Pd(OAc)_2_ to form a five-membered palladacycle A, which undergoes oxidative addition of iodobenzene (117) to form the Pd^IV^ species B. Next, B suffers a reductive elimination to give the arylated intermediate C, regenerating the catalyst.

Subsequent deprotonation of the amine in C and C–H activation affords the seven-membered palladacycle D,^148^ which undergoes a reductive elimination to furnish 118 and a Pd^0^ species. The latter is finally oxidized by Ag_2_CO_3_ to regenerate the active Pd^II^ species for the next catalytic cycle.

#### Synthesis by Mhaske *et al.*

**Scheme 28:**
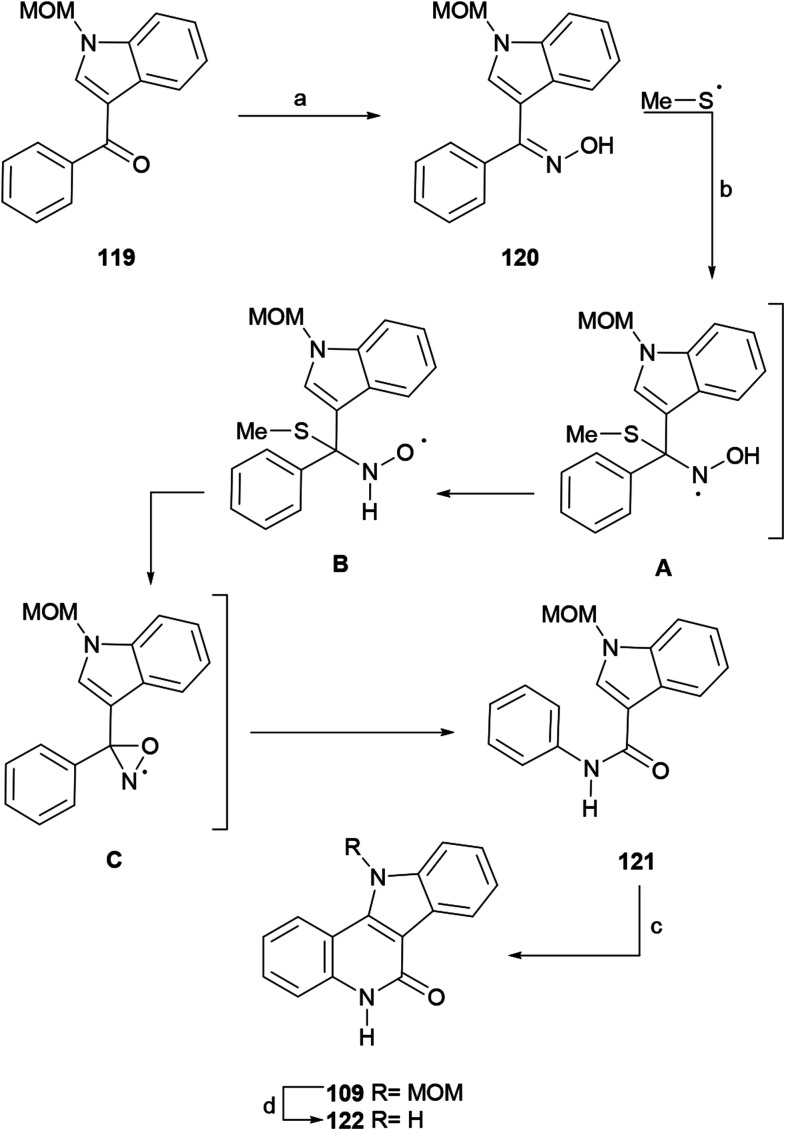
Reagents and conditions: (a) NH_2_OH·HCl, NaOAc, MeOH : H_2_O (10 : 3, v/v), reflux, 24 h (88%); (b) (NH_4_)_2_S_2_O_8_, DMSO, dioxane, 6 h (51%); (c) Pd(OAc)_2_, Cu(OAc)_2_, AcOH, 18 h (66%); (d) 4 N HCl, dioxane, 4 h (95%).

Mhaske and coworkers reported the application of (NH_4_)_2_S_2_O_8_–DMSO as an inexpensive, environmentally benign and safe reagent under mild neutral conditions, for the synthesis of imides, methylene bis-amides, diarylmethanes, and also for the Mannich reaction.^149^ The usefulness of the developed protocol was demonstrated by a formal total synthesis of isocryptolepine (Scheme 28).^150^

The synthetic sequence commenced with 3-benzoylindole,^151^ which in the successful synthetic sequence was protected with a MOM group to afford 119, since electron withdrawing protecting groups (benzoyl, tosyl) failed during more advanced stages. In turn, 119 was converted to oxime 120 in 88% yield under conventional conditions, and the latter was subjected to the standard oxidation protocol to furnish the radical Beckmann rearrangement product, the amide 121, in moderate yield (51%).

A mechanism for this rearrangement was proposed. It was suggested that the (NH_4_)_2_S_2_O_8_-DMSO system is a source of methylsulfanyl radicals (MeS˙), which can attack the oxime moiety of 120 and afford the radical intermediate A. The latter can undergo a rearrangement to B and be further converted into the oxaziridine C,^152^ with the loss of MeSH.

In turn, the latter intermediate finally undergoes a rearrangement to afford amide 121. Radical intermediates have also been observed in the photochemically driven Beckmann rearrangement.^153^

The as-formed intermediate 121 was exposed to Pd(OAc)_2_ and Cu(OAc)_2_ in AcOH at 120 °C, which promoted an intramolecular double C–H activation, affording 109 in 66% yield. A similar C–C bond formation under UV light irradiation (254 nm) was reported earlier.^154^ Compound 109 is a key intermediate in Hibino's synthesis of isocryptolepine;^134^ therefore, this sequence constitutes a formal total synthesis of isocryptolepine. Additionally, compound 109 was deprotected with HCl in dioxane to obtain indoloquinolinone 122, a well-known bioactive scaffold, in 95% yield.^147*a*,155^

#### Synthesis by Zhang *et al.*

The Zhang group developed several copper-mediated syntheses of natural products and in 2018 reported a selective cascade C–H/N–H annulation strategy toward the tetracyclic core of isocryptolepine, thus providing a new formal total synthesis of the natural product.^156^ The bond forming approach is mediated by a 8-aminoquinoline *N*,*N*-bidentate directing group^157^ and involves a reaction with a benzyne derivative under mild conditions.

The general transformation was optimized to use 0.35 equiv. of Cu(OAc)_2_ and 1.2 equiv. CsF as a base, in the presence of 0.5 equiv. of TBAI, which enhances the generation of benzyne at 80 °C in DMF : MeCN (1 : 1, v/v). The reaction took place under oxygen as a final oxidant, for 12 h. The synthetic sequence leading to the isocryptolepine precursor involved the use of 5-methoxy-8-aminoquinoline as a removable sacrificial *N*,*N*-ligand^158^ and the Kobayashi benzyne precursor 124.^159^

**Scheme 29:**
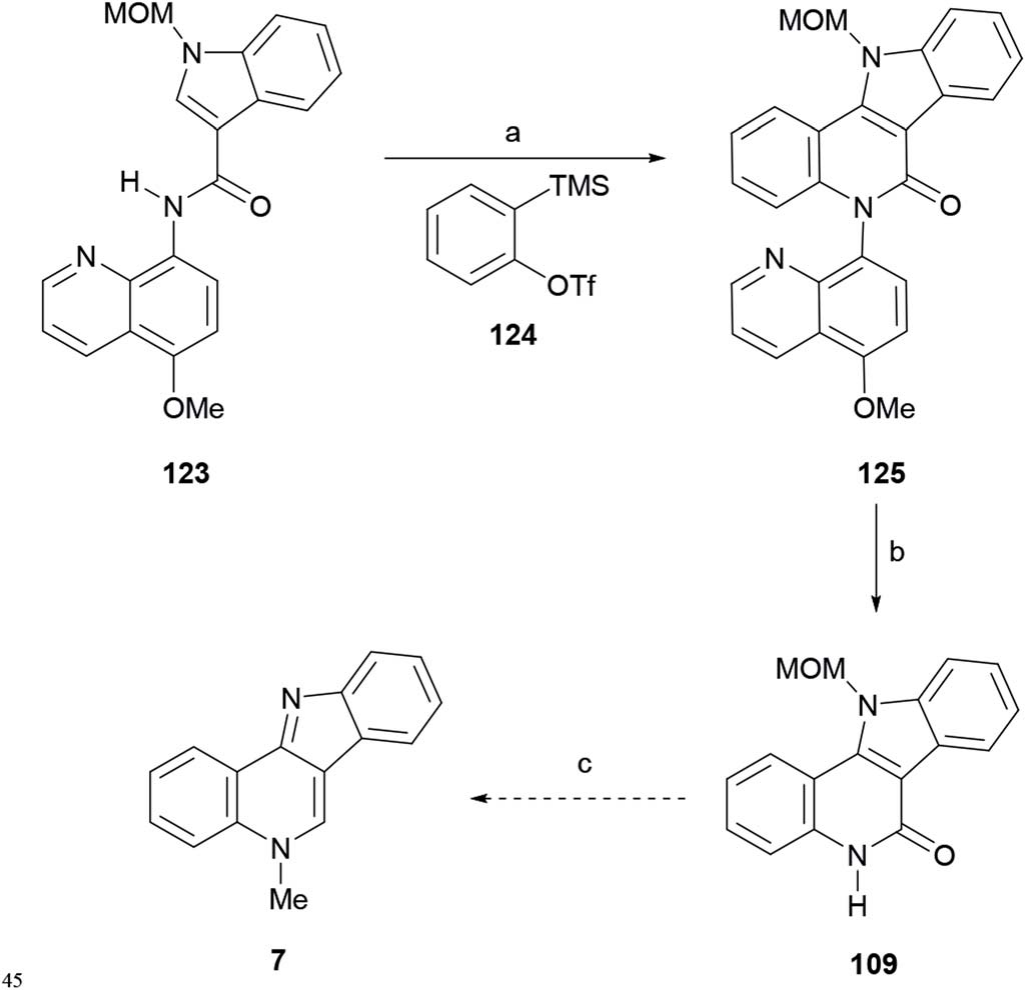
Reagents and conditions: (a) 124, Cu(OAc)_2_, CsF, TBAI, DMF : MeCN (1 : 1, v/v), O_2_, 80 °C, 12 h (62%); (b) (1) BBr_3_, CH_2_Cl_2_ (67%); (2) K_2_Cr_2_O_7_, AcOH : H_2_O (1 : 1, v/v) (65%); (c) see ref. 134.

In the synthetic sequence, 124 was induced to react with the indole-3-carboxamide derivative 123 to afford 125 in 62% yield (Scheme 29), carrying a removable MOM group.

Then, the quinoline auxiliary was removed *via* a two-step protocol, including the selective *O*-demethylation of the quinolyl methyl ether with BBr_3_ to free the phenol group, which was further oxidized with K_2_Cr_2_O_7_ in AcOH : H_2_O (1 : 1, v/v), to afford lactam 109 in 65% yield, Hibino's intermediate toward isocryptolepine.^134^

This reaction system avoids the use of sensitive and expensive noble metals and oxidants, and complements previous metal-based strategies.

**Scheme 30:**
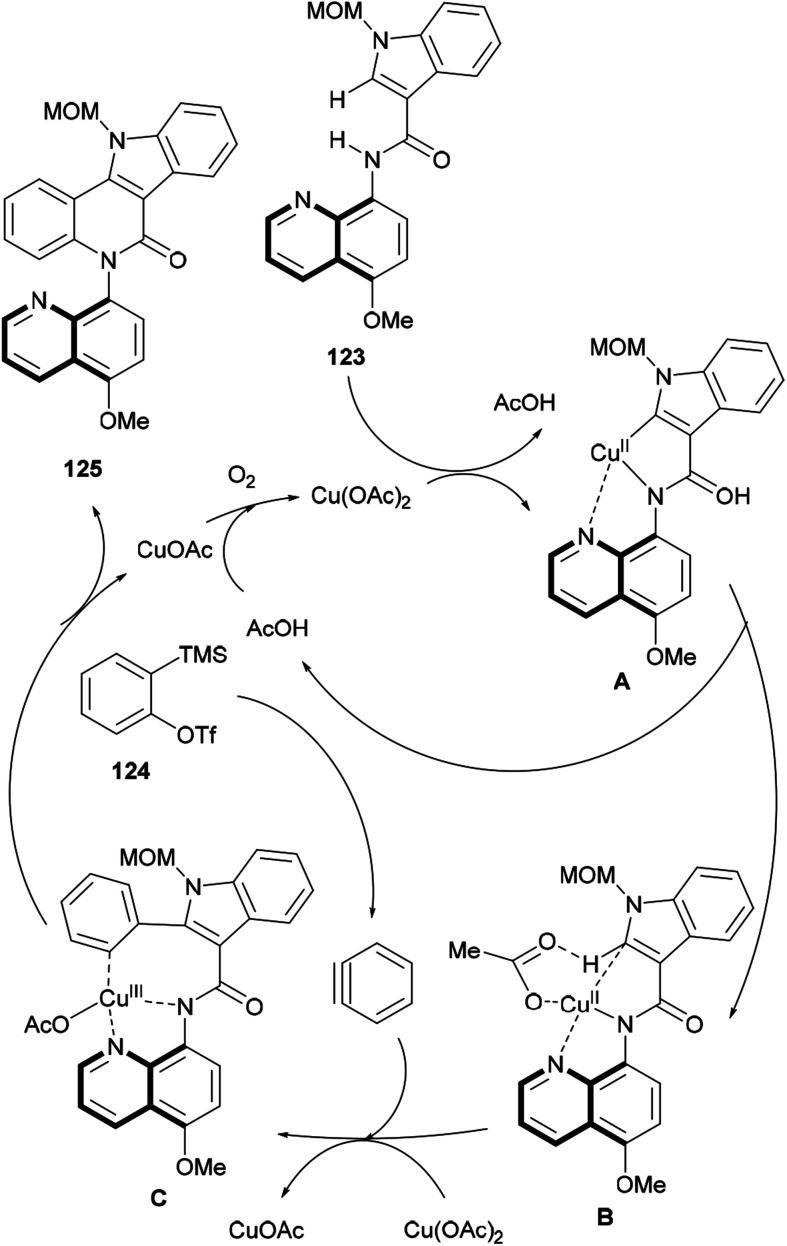
Proposed reaction mechanism for the copper(ii)-mediated selective cascade C–H/N–H annulation strategy.

A plausible mechanism for this C–H/N–H cascade annulation was presented, where a Cu(iii)-mediated organometallic C–H activation takes place (Scheme 30).^160^ Thus, 123 coordinates with Cu(OAc)_2_ to generate an anionic complex A ligated by a *N*,*N*-bidentate directing group.^161^ Then, the complex undergoes acetate-assisted intramolecular C–H concerted metalation/deprotonation to afford the five-membered ring complex B, which then undergoes carbocupration with the aryne generated from precursor 124 to furnish the intermediate C. The final reductive elimination of CuOAc C provides the desired annulation product (125), with the catalyst being regenerated for the next cycle in the presence of oxygen.

### 4e. Summary of the synthetic efforts toward isocryptolepine

In general, the reported synthetic routes toward isocryptolepine required multistep linear sequences with the corresponding isolation and purification stages of the intermediate products. A comparison of them (Table 1), in terms of complexity and overall yield, is difficult and not straightforward, due to the variable nature and structural complexity of the starting materials. In addition, many syntheses of isocryptolepine are actually formal total syntheses, reaching only a previously known advanced intermediate toward the natural product.

**Table 1:** Chronological summary of the total syntheses and relevant formal total syntheses of isocryptolepine

Year	Group	Class^a^	No. of steps	Main reaction(s) in the strategy	Yield (%)	Ref.
1950	Kermack	Q	4	Graebe–Ullmann of 4-benzotriazolylquinoline	29.5	43
1955	Mann	Q	3	Fischer indolization of *N*-methyl tetrahydroquinolin-4-one	<15	45*a*
1993	Alvarez-Builla	Q	2^b^	Graebe–Ullmann of 4-benzotriazolylquinoline	54	49
1996	Novikov	B	6	Fischer indolization of *N*-tosyl tetrahydroquinolin-4-one	15.5	50
1997	Tímari	Q	5	Suzuki cross-coupling; nitrene cyclization	46.7	97
1998	Joule	I	3	Suzuki coupling; Vilsmeier closure	45.6	122
1999	Molina	B	11	Cyanate and nitrene cyclizations	24.6	54*a*
2002	Mohan	I	3	Photochemical ring closure	47.3	89*a*
2003	Maes	Q	3	Buchwald–Hartwig; intramolecular direct arylation	43.9	104
2005	Mohan	Q	3	Fisher indolization of *N*-methyl tetrahydroquinolin-2,4-dione	45.5	114
2006	Mohan	Q	3	Heteroatom directed photoannulation	64.7	10*b*
2007	Miki	I	4	Decarboxylative Heck-cyclization	30.2	129
2008	Maes	Q	2	Auto-tandem Pd-catalyzed consecutive C–N and C–C bond formation	46.7	112
2009	Kundu	B	2^b^	Modified Pictet–Spengler	86	59
2009	Kumar	Q	1	Fisher indolization; oxidation of *N*-methyl tetrahydroquinolin-4-one	83	120
2010	Kraus	B	5	Electrocyclization of the anion of a Schiff base	47.8	68
2010	Kraus	B	4^c^	Intramolecular Wittig reaction	60.8	69
2011	Kusurkar	I	6	6π-Electrocyclization	37.1	132
2012	Butin	B	4	Furan to indole recyclization; Michael addition	11.6	73
2012	Hibino	I	9	Curtius rearrangement; 6π-electrocyclization	53.6	134
2012	Tummatorn	I	4	Aza-[4 + 2]-cycloaddition	65.5	139
2013	Bogányi	B	7^b^	Buchwald–Hartwig amination; Heck-type biaryl coupling	21.8	76
2014	Lin	I	3	Pd(ii)-Catalyzed direct dehydrogenative annulation	43.3	144
2016	Lu & Xu	B	4	Electrosynthesis. Cyclization of acetylenic acetal *via* amidyl radicals	36.2	85
2016	Mhaske	I	4^d^	Radical Beckmann rearrangement; Pd-catalyzed double C–H activation	29.6	150
2017	Aksenov & Rubin	B	2	Fischer indolization; Vilsmeier reaction with triazine	83	90
2018	Zhang	I	2^e^	Selective cascade C–H/N–H annulations	27	156

a Classification according to the structure of the starting material. B = benzenoid; Q = quinoline; I = indole.

b Formal total synthesis. Data correspond to the synthesis of the unsubstituted tetracycle 1.

c Formal total synthesis. Data correspond to the synthesis of the azide 36, an intermediate in the synthesis of Molina *et al.*

d Formal total synthesis. Data correspond to the synthesis of 109, the tetracyclic intermediate of Hibino.

e Formal total synthesis. Data correspond to the synthesis of 109, the tetracyclic intermediate of Hibino.

However, it is worth noting that several very short approaches, consisting of just one to three steps using commercially available materials, have been reported, and various approaches have allowed access to the natural product in more than 60% overall yield.

## 5. Conclusions and perspective

The indoloquinoline heterocyclic system is strongly associated with the West African plant *Cryptolepis sanguinolenta*, although other natural sources are known. Cryptosanguinolentine (isocryptolepine) has been isolated twice from this plant, widely used in traditional medicine.

Its unique framework and interesting pharmacological properties have meant that over the last 20 years isocryptolepine has become a test ground for new synthetic approaches toward C–C and C–N bond formation, with or without the use of precious metal catalysts. The natural product has been the target of over 25 synthetic efforts, where the tetracycle was built from benzenoids, quinolines and/or indoles *via* the stepwise construction of the heterocyclic moieties or through the successive assembly of the remaining heterocyclic rings.

The natural product has also gained recognition as an important and privileged scaffold in drug discovery due to the broad spectrum of its biological activities. Hence, many of the developed strategies have proved to be useful for the synthesis of more complex analogs and derivatives of the natural product, embodied with more potent and/or selective activity, mainly as cytotoxic and antimalarial agents. Therefore, it can be foreseen that the virtuous cycle, which involves new chemistries and novel structures for improved bioactive compounds, will keep going in the future, meaning that novel total syntheses of isocryptolepine will keep being disclosed.

## Conflicts of interest

There are no conflicts to declare

## Supplementary Material
